# Zero-profile implant versus conventional cage–plate construct in anterior cervical discectomy and fusion for the treatment of single-level degenerative cervical spondylosis: a systematic review and meta-analysis

**DOI:** 10.1186/s13018-022-03387-9

**Published:** 2022-11-24

**Authors:** Alafate Kahaer, Ruilin Chen, Muzaipaer Maitusong, Peierdun Mijiti, Paerhati Rexiti

**Affiliations:** 1 Department of Spine Surgery, The First Affiliated Hospital of Xinjiang Medical University, Urumqi, 830054 Xinjiang Uygur Autonomous Region China; 2 Xinjiang Medical University, Urumqi, China; 3 School of Public Health, Xinjiang Medical University, Urumqi, 830054 Xinjiang Uygur Autonomous Region China

**Keywords:** Single level, Zero-profile, Anterior cervical discectomy and fusion, ACDF, Clinical outcome

## Abstract

**Background:**

The clinical outcomes of single-level anterior cervical discectomy and fusion (ACDF) with the Zero-profile (Zero-p) were evaluated in comparison with the anterior cervical cage–plate construct (CPC).

**Methods:**

We performed a systematic search covering PubMed, Embase, Cochrane Central Register of Controlled Trials, Web of Science, Medline, China National Knowledge Infrastructure (NCKI), Wan Fang Database, and Wei Pu Database. Articles focused on single-level ACDF or data of the single - level that can be extracted were included, and articles that did not directly compare Zero-p and CPC were excluded. Twenty-seven studies were included with a total of 1866 patients, 931 in the Zero-p group and 935 in the CPC group. All outcomes were analyzed using Review Manager 5.4.

**Results:**

The meta-analysis outcomes indicated that operative time (WMD = − 12.47, 95% CI (− 16.89, − 8.05), *P* < 0.00001), intraoperative blood loss (WMD = − 13.30, 95% CI (− 18.83, − 7.78), *P* < 0.00001), risk of adjacent segment degeneration (ASD) (OR 0.31, 95% CI (0.20, 0.48), *P* < 0.0001), risk of dysphagia of short-term (OR 0.40, 95% CI (0.30, 0.54), *P* < 0.0001), medium-term (OR 0.31, 95% CI (0.20, 0.49), *P* < 0.0001), and long-term (OR 0.29, 95% CI (0.17, 0.51), *P* < 0.0001) of Zero-p group were significantly lower. The JOA score of Zero-p group at the final follow-up was significantly higher (WMD = − 0.17, 95% CI (− 0.32, − 0.03), *P* = 0.02). There were no significant differences in length of stay (LOS), Neck Disability Index (NDI), Visual Analogue Score (VAS), fusion rate, segmental Cobb angle, cervical Cobb angle, prevertebral soft tissue thickness (PSTT), SF-36, subsidence, implant failure, and hoarseness between the two groups. This study was registered with PROSPERO, CRD42022347146.

**Conclusion:**

Zero-p group reduced operative time, intraoperative blood loss, JOA score at follow-up and reduced the incidence of dysphagia and postoperative ASD, but the two devices had the same efficacy in restoring the cervical curvature, preventing the cage subsidence, and in postoperative VAS, NDI, LOS, PSTT, SF-36, fusion rate, implant failure, and hoarseness in single-level ACDF. The use of Zero-p in single-level ACDF was recommended.

**Supplementary Information:**

The online version contains supplementary material available at 10.1186/s13018-022-03387-9.

## Background

The number of patients who need surgical treatment with degenerative cervical spondylosis (DCS) has increased in recent years [1]. Since it was introduced by Cloward in 1958 [2], anterior cervical discectomy and fusion (ACDF) has been the gold-standard surgical technique for both single- and multi-level DCS [3]. The anterior cage and plate construct (CPC), which can be utilized for single- and multi-level cervical spondylosis, is a commonly adopted surgical device. Its superior stability, decompression rate, and fusion rate have been endorsed in great amount of studies; therefore, it is widely used in clinical practice [4]. However, complications such as a higher incidence rate of postoperative dysphagia and adjacent segment degeneration (ASD) have been documented [5, 6].

A Zero-profile interbody spacer (Zero-p) is presently being utilized in clinical trials to reduce the risk of the aforementioned complications. The Zero-p, unlike the CPC, can be inserted into the intervertebral space without the necessity for an extra titanium plate in front of the vertebral body. It has been proven in several studies to greatly reduce the incidence of postoperative dysphagia and ASD [7–10]. This may be due to its integrated design, which does not protrude the front rim of the cervical vertebrate [11]. However, the literature shows that the Zero-p cannot effectively maintain intervertebral height and cervical curvature after the surgery when compared to the CPC [12]. According to the biomechanical study of Li et al. [13], the range of motion (ROM) and maximum stress of the Zero-p were lower than those of CPC.

Recently, several studies have compared the clinical and radiological outcomes of Zero-p and CPC in ACDF for treating multi-level DCS. However, there was no meta-analysis comparing the Zero-p and CPC in single-level ACDF with complete outcomes was found. The goal of this study is to compare the clinical and radiological results of the Zero-p and CPC in ACDF for single-level DSC to provide complete evidence to support the use of Zero-p in the single-level ACDF.

## Methods

### Literature search

Our research follows the Preferred Reporting Items for Systematic Reviews and Meta-Analysis (PRISMA) standards [14, 15]. Two independent investigators (Kahaer and Chen) investigated electronic databases or platforms (PubMed, Embase, Cochrane Central Register of Controlled Trials, Web of Science, Medline, NCKI, Wan Fang Database, and Wei Pu Database). The search was conducted with the following searching strategy as follows: “zero profile,” “Zero-profile,” “Zero-p,” “zero p,” “no-profile,” “anchored,” “ROI-C,” “self-locking,” “ACDF,” “anterior cervical discectomy and fusion” with various combinations of the “AND,” “NOT,” and “OR.” We restricted the language to English and Chinese. By preserving the literature that offered the most comprehensive information for overlapping patients, information duplication was avoided.

### Selection criteria

The inclusion criteria were as follows: (1) the literature compared patients with DSC who underwent the single-level ACDF using Zero-p and CPC and (2) the literature reported one of the followings: operative time, Intraoperative blood loss, length of stay (LOS), Neck Disability Index (NDI), Japanese Orthopaedic Association (JOA) score, Visual Analogue Score (VAS), prevertebral soft tissue thickness (PSTT), 36-Item Short Form Survey (SF-36), segmental and cervical Cobb angle, fusion rate, adjacent segment degeneration (ASD), cage subsidence, dysphagia, implant failure, hoarseness.

The exclusion criteria were as follows: (1) There was no evidence of the Zero-p or CPC or ACDF, (2) literature reviews, meeting abstracts, pathology reports, conference reports, editorials, expert opinions, animal trials, autopsies, meta-analyses, case reports, biomechanical studies, and other associated investigations, (3) the literature in which data cannot be extracted, (4) presence of infection, tumor, history of previous cervical spine surgery, severe ossification of the posterior longitudinal ligament, and (5) the literature of two or multi-level ACDF.

### Quality assessment and data extraction

Using a predesigned data extraction sheet, pairs of authors (Kahaer and Chen) independently extracted data from the included literature. Non-randomized controlled studies used Newcastle–Ottawa Scale (NOS) to evaluate the quality. A maximum of 9 points and greater than 6 were considered the high-quality literature. Randomized controlled trials (RCTs) used the Delphi list to evaluate the quality. Two authors independently assessed the quality of each study and then cross-checked, with a third evaluator (Maitusong) handling any disagreements. Authors, publication date, title, study design, indications, fusion levels, follow-up time, number of patients, mean age of patients, design of the Zero-p device, and clinical outcomes were extracted from the qualified literature. This study was registered with PROSPERO, CRD42022347146.

### Statistical analysis

Data analysis was performed using Review Manager Software (RevMan 5.4, The Cochrane Collaboration). Continuous data including operative time, intraoperative blood loss, LOS, NDI, JOA, VAS, PSTT, SF-36, segmental Cobb angle, and cervical Cobb angle were analyzed using weighted mean differences (WMD) and 95% confidence intervals (CI). Dichotomous outcomes including fusion, adjacent segment degeneration (ASD), cage subsidence, dysphagia, implant failure, and hoarseness were analyzed using the odds ratio (OR). Heterogeneity between studies was tested using the I^2^ statistic. When the *I*^2^ > 50% (high heterogeneity), a random effect model was used. If it was ≤ 50% (low heterogeneity), a fixed-effect model was used. A funnel plot was also used to assess publication bias. *P* < 0.05 was considered statistically significant.

## Results

### Literature search

**Fig. 1:**
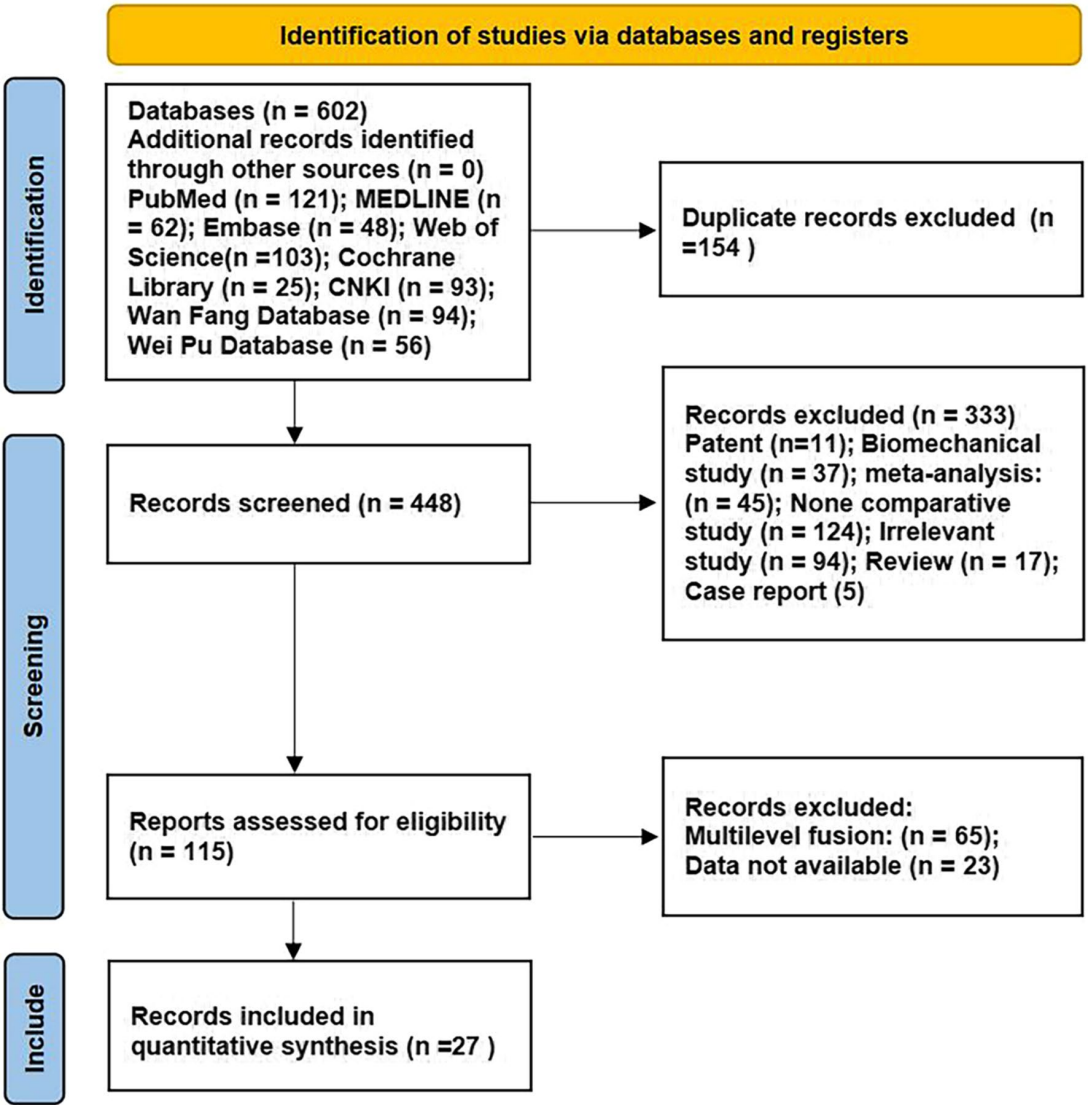
Flow diagram of study selection

There were 602 studies which were searched from 8 electronic databases (PubMed, *n* = 121; Embase, *n* = 48; Cochrane Central Register of Controlled Trials, *n* = 25; Web of Science, *n* = 103; Medline, *n* = 62; NCKI, *n* = 93; Wan Fang Database, *n* = 94; Wei Pu Database, *n* = 56). Of these, 154 were duplicates and 448 were excluded after the title and abstract screening. After careful full-text evaluation, as a result, 27 studies including English and Chinese were included [7–11, 16–37] and data were extracted. A flow diagram of the literature searching strategy is shown in Fig. 1.

### Literature characteristic and quality assessment

Four prospective RCTs [20, 22, 26, 30] and 23 retrospective observational literature [7, 8, 10, 11, 16–19, 21, 23–25, 27–29, 31–38] were included. A total of 931 patients with Zero-p and 935 patients with CPC were compared. The design of the Zero-p devices was as follows: Zero-p (DePuy, Synthes, USA), Zero-p (Synthes GmbH, Oberdorf, Switzerland), and Zero-p (Synthes, Zuchwil, Switzerland), ROI-C, and PREVAIL (Medtronic Sofamor Danek, Memphis, TN, USA). The differences in the patient’s age, BMD, BMI, and follow-up time were not significant (Additional file 1). The basic characteristics and demographics are presented in Table 1. The description of clinical features is presented in Table 2. In terms of quality assessment, NOS was used for the non-randomized controlled trials. The scores of all included literature covered 6–9 points, defined as high quality, as shown in Table 3. Quality assessment of RCTs based on the Delphi list is given in Table 4.

**Table 1:** Study characteristics and demographics

References	Year	Study design	Sample size		Mean age		Operation Time (min)		Blood Loss (mL)	
			Zero-p	CPC	Zero-p	CPC	Zero-p	CPC	Zero-p	CPC
Lan et al. [7]	2017	R OS	35	33	54.05 ± 10.11	52.09 ± 10.46	101.57 ± 14.36	107.88 ± 14.35	93.4 ± 9.04	97.94 ± 10.76
Vaishnav et al. [8]	2018	R OS	41	23	48.58 ± 10.72	46.37 ± 8.4	44.88 ± 6.54	54.43 ± 14.71	27.32 ± 9.23	30.68 ± 13.21
Wei et al. [11]	2022	R OS	68	28	48.7 ± 7.3	47.2 ± 6.7	55.24 ± 5.17	53.16 ± 5.29	34.52 ± 6.42	32.46 ± 8.31
He et al. [16]	2021	R OS	42	45	62.59 ± 8.21	61.15 ± 7.52	84 ± 23	98 ± 27	139 ± 22	154 ± 33
Noh et al. [17]	2021	R OS	38	42	51.9 ± 10.21	52.6 ± 8.61	108.31 ± 17.15	123.25 ± 28.23	72.39 ± 13.11	92.12 ± 38.65
Lee et al. [18]	2015	R OS	23	18	57.26 ± 13.28	52.89 ± 7.71	NS	NS	NS	NS
Son et al. [19]	2014	R OS	21	27	55.4 ± 9.7	50.2 ± 10.9	159.5 ± 52.1	147.4 ± 48.4	90 ± 148	146.5 ± 138.0
Nemoto et al. [20]	2015	P RCT	24	22	40.9 ± 7.2	41.6 ± 7.0	116.4 ± 17.1	128.5 ± 17.4	NS	NS
Wang et al. [21]	2014	R OS	22	25	50.86 ± 8.79	53.68 ± 8.96	98.18 ± 15.55	105.4 ± 14.43	87.95 ± 12.02	92.4 ± 11.28
Li et al. [22]	2015	P RCT	23	23	NS	NS	NS	NS	NS	NS
Wang et al. [23]	2015	R OS	27	30	51.6 ± 11.3	54 ± 8.5	98.2 ± 15.2	109.8 ± 16.9	88.2 ± 12.9	95.2 ± 11.6
Yan et al. [24]	2014	R OS	37	35	63.55 ± 7.12	64.28 ± 8.76	76.59 ± 14.53	53.78 ± 17.91	52.74 ± 26.84	85.46 ± 23.97
Li et al. [25]	2020	R OS	24	27	65.7 ± 7.5	62.3 ± 3.4	NS	NS	NS	NS
Liu et al. [26]	2016	P RCT	31	31	48.5 ± 9.1	45.2 ± 10.6	63.45 ± 10.87	85.97 ± 12.04	44.35 ± 11.53	66.26 ± 19.62
Shao et al. [27]	2016	R OS	63	76	47.6 ± 6.4	50.3 ± 8.2	63.7 ± 12.5	71.8 ± 13.2	83.6 ± 14.5	86.1 ± 14.3
Yi et al. [28]	2017	R OS	80	84	52.12 ± 5.893	51.95 ± 6.267	148.46 ± 27.239	165.37 ± 28.538	76.87 ± 21.38	80.46 ± 31.409
Wang et al. [29]	2016	R OS	12	16	50.5 ± 13.5	52.0 ± 12.0	113 ± 8.6	160.44 ± 17.2	51.67 ± 24.2	52.13 ± 24.54
Guo et al. [30]	2015	P RCT	49	49	43.1 ± 16.9	43.3 ± 17.7	70.8 ± 17.3	87.6 ± 23.4	49.5 ± 17.2	65.2 ± 25.3
Sun et al. [31]	2017	R OS	25	28	53 ± 10.26	53.57 ± 10.66	88.88 ± 25.8	109.2 ± 20.4	83.22 ± 33.24	117.33 ± 23.57
Hu et al. [32]	2017	R OS	23	31	49.78 ± 10.4	45.54 ± 40.22	78.22 ± 14.01	82.19 ± 6.51	19.52 ± 6.95	37.16 ± 5.25
Sha et al. [33]	2021	R OS	30	31	52.9 ± 9.27	50.33 ± 8.57	81.33 ± 10.74	93.67 ± 11.59	77.33 ± 22.43	108.33 ± 24.08
Chang et al. [9]	2017	R OS	21	24	54.6 ± 3.5	53.2 ± 4.2	62.7 ± 17.3	87.6 ± 23.2	78.4 ± 29.6	80.2 ± 36.8
Ruan et al. [34]	2018	R OS	21	18	56.3 ± 9.8	59.6 ± 12.5	68.6 ± 8.2	79.7 ± 9.3	41.2 ± 7.4	78.9 ± 9.2
Zhu et al. [35]	2019	R OS	19	26	55.42 ± 8.03	59.15 ± 8.04	129 ± 25	172 ± 29	88 ± 29	151 ± 33
Zhang et al. [36]	2020	R OS	56	67	45.2 ± 13.9	48.7 ± 13.2	76.96 ± 8.98	82.31 ± 7.57	51.64 ± 20.35	57.97 ± 17.9
Gou et al. [10]	2022	R OS	16	16	48.5 ± 6.7	52.4 ± 7.2	75.1 ± 6.0	90.6 ± 8.1	61.9 ± 9.9	60.3 ± 12.6
Luo et al. [37]	2021	R OS	60	60	59.1 ± 16.4	59.8 ± 14.4	75.22 ± 7.57	90.39 ± 8.1	77.53 ± 37.27	72.24 ± 34.74

R, retrospective; P, prospective; OS, observational; RCT, randomized controlled trial; and NS, not specified

**Table 2:** Description of clinical features of studies

Study	Indication (s)	Design of Zero-profile device	Fusion level (Zero-p/CPC)					Mean follow-up time (month)	
			C3/4	C4/5	C5/6	C6/7	C7/T1	Zero-p	CPC
Lan et al. [7]	CR, CSM	Zero-p	8/7	10/12	13/11	4/3		23.68 ± 1.93	24.39 ± 2.00
Vaishnav et al. [8]	NS	Zero-p	2/1	2/3	8/5	12/13		NS	NS
Wei et al. [11]	CR, CSM	Zero-p	5/6	8/21	15/32	9/9		15.3 ± 5.2	15.1 ± 5.2
He et al. [16]	CR, CSM	ROI-C						22.6 ± 3.3	27.1 ± 3.5
Noh et al. [17]	CR	Zero-p		8/8	19/24	11/14		37.6 ± 5.91	37.1 ± 15.7
Lee et al. [18]	CR	Zero-p	4/1	2/4	13/9	4/4		12.57 ± 2.09	28.89 ± 20.24
Son et al. [19]	CR	Zero-p	3/2	4/6	10/14	4/5		≥ 6	≥ 6
Nemoto et al. [20]	CR	PREVAIL	2/2	4/6	10/10	6/6		24	24
Wang et al. [21]	CSM	Zero-p						33.59 ± 5.52	33.16 ± 5.97
Li et al. [22]	CR, CSM	Zero-p	11/9	9/11	3/3			24	24
Wang et al. [23]	CSM	Zero-p	2/3	8/7	9/12	8/8		35.2	35.5
Yan et al. [24]	CR, CSM	Zero-p			12/13	25/22		15.32 ± 2.13	14.26 ± 2.35
Li et al. [25]	CR, CSM	Zero-p	5/7	10/10	9/10			81.0 ± 4.4	79.0 ± 3.4
Liu et al. [26]	CSM	Zero-p	3/2	9/13	13/11	6/5		15.52 ± 1.93	16.10 ± 2.33
Shao et al. [27]	NS	Zero-p	2/4	24/27	31/36	6/9		23.6 ± 4.5	25.2 ± 4.8
Yi et al. [28]	CR, CSM	Zero-p						> 12	> 12
Wang et al. [29]	CR, CSM	Zero-p		2/5	6/8	4/6		NS	NS
Guo et al. [30]	CR, CSM	Zero-p	13/10	9/13	9/9	10/12	9/5	18.5 ± 17.5	18.5 ± 17.5
Sun et al. [31]	CR, CSM	ROI-C						3–24	3–24
Hu et al. [32]	CR, CSM	ROI-C						15.7 ± 2.4	15.7 ± 2.4
Sha et al. [33]	CR, CSM	ROI-C	8/7	10/11	12/13			13.5 ± 1.5	13.5 ± 1.5
Chang et al. [9]	CSM	Zero-p	3/4	10/12	5/5	3/3		12–16	12–16
Ruan et al. [34]	CSM	ROI-C	0/1	2/4	12/8	7/5		13.3 ± 1.9	14.9 ± 1.7
Zhu et al. [35]	CR, CSM	ROI-C						> 12	> 12
Zhang et al. [36]	CR, CSM	ROI-C	7/11	17/23	23/25	9/8		21.46 ± 4.51	21.46 ± 4.51
Gou et al. [10]	CSM	Zero-p	2/1	5/4	7/6	2/5		6–18	6–18
Luo et al. [37]	CR	Zero-p	5/3	9/10	23/22	13/15		≥ 24	≥ 24

CR = cervical radiculopathy, CSM = cervical spondylotic myelopathy, NS = not specified

**Table 3:** Quality assessment using the Newcastle–Ottawa quality assessment scale for each non-randomized controlled trial

Variable	Lan et al. [7]	Vaishnav et al. [8]	Wei et al. [11]	He et al. [16]	Noh et al. [17]	Lee et al. [18]	Son et al. [19]	Wang et al. [21]	Wang et al. [23]	Yan et al. [24]	Li et al. [25]	Shao et al. [27]	Yi et al. [28]	Wang et al. [29]	Sun et al. [31]	Hu et al. [32]	Sha et al. [33]	Chang et al. [9]	Ruan et al. [34]	Zhu et al. [35]	Zhang et al. [36]	Gou et al. [10]	Luo et al. [37]
*Selection*																							
Representativeness of exposed cohort	1	1	1	1	1	1	1	1	1	1	1	1	1	1	1	1	1	1	1	1	1	1	1
Selection of non-exposed cohort	1	1	1	1	1	1	1	1	1	1	1	1	1	1	1	1	1	1	1	1	1	1	1
Ascertainment of exposure	1	1	1	1	1	1	1	1	1	1	1	1	1	1	1	1	1	1	1	1	1	1	1
Demonstration that outcome of interest was not present at start of study		1	1	1								1	1							1			
*Comparability*																							
Study controlled for age or gender	1	1	1	1	1	1	1	1	1	1	1	1	1	1	1	1	1	1	1	1	1	1	1
Study controlled for any additional factor	1	1	1	1	1	1	1	1	1	1	1	1	1		1	1		1	1	1	1	1	1
*Outcome*																							
Assessment of outcome	1	1	1	1	1	1	1	1	1	1	1	1	1	1	1	1	1	1	1	1	1	1	1
Follow-up long enough for outcomes to occur	1	1	1	1	1	1	1	1	1	1	1	1	1	1	1	1	1	1	1	1	1	1	1
Adequacy of follow-up of cohort	1		1				1	1	1	1	1	1	1		1	1	1	1	1	1	1	1	1
Total	8	8	9	8	7	7	8	8	8	8	8	9	9	6	8	8	7	8	8	9	8	8	8

**Table 4:** Methodological quality assessment of included randomized controlled trials using Delphi list

Variable	Study			
	Nemoto et al. [20]	Li et al. [22]	Liu et al. [26]	Guo et al. [30]
Randomization method used	Yes	Yes	Yes	Yes
Groups were similar at baseline regarding most important prognostic indicators	Yes	Yes	Yes	Yes
Eligibility criteria were specified	Yes	Yes	Yes	No
Outcome assessor was blinded	No	No	Yes	No
Care provider was blinded	No	No	No	No
Patient was blinded	No	Yes	No	No
Point estimates and measures of variability were presented for primary outcome measures	Yes	Yes	Yes	Yes
Analysis includes an intention-to-treat analysis	No	No	No	No

### Clinical outcomes

#### Operative time

**Fig. 2:**
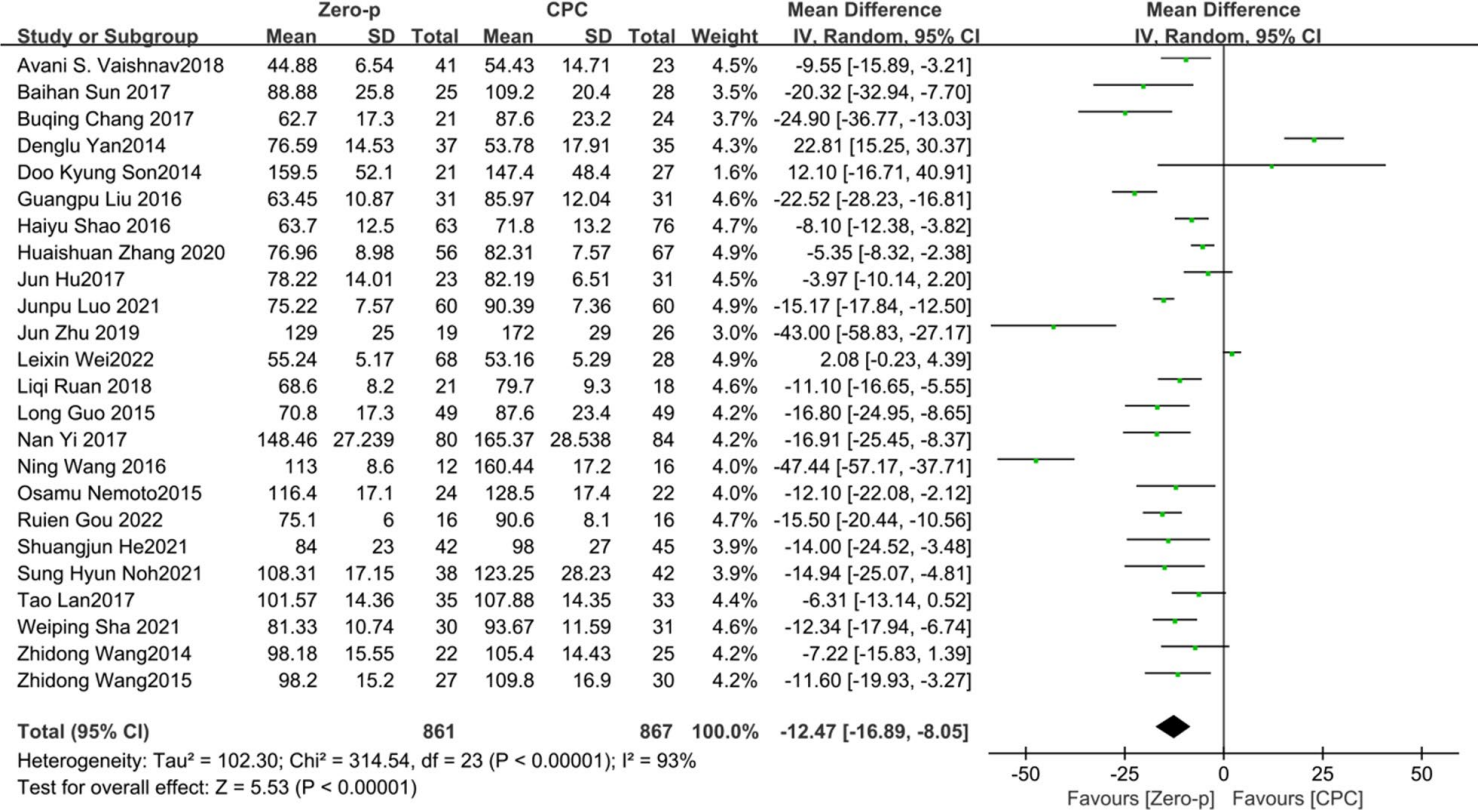
Meta-analysis of Zero-p group versus CPC group in operative time

24 studies [7–11, 16, 17, 19–21, 23, 24, 26–37] consisting of 1728 patients (Zero-p group, 861; CPC group, 867) compared the mean operative time. There was significant heterogeneity in the literature (*P* < 0.00001, *I*^2^ = 93%). Meta-analysis was performed using random-effect model, and the result showed that operative time in the CPC group was significantly greater than that of in Zero-p group (WMD = − 12.47, 95% CI (− 16.89, − 8.05), *P* < 0.00001). The corresponding forest plot was shown in Fig. 2.

#### Intraoperative blood loss

**Fig. 3:**
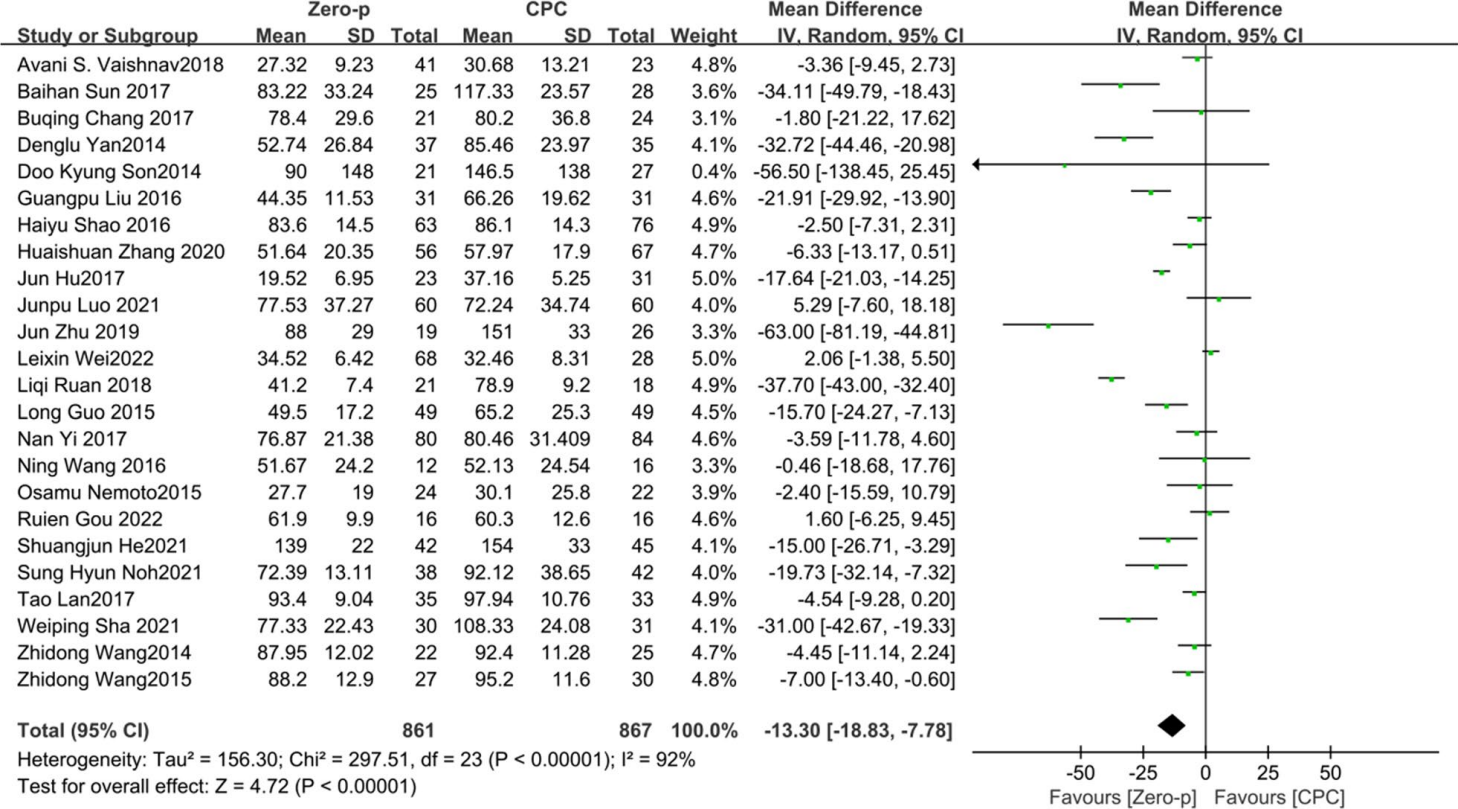
Meta-analysis of Zero-p group versus CPC group in intraoperative blood loss

Studies [7–11, 16, 17, 19–21, 23, 24, 26–37] consisting of 1728 patients (Zero-p group, 861; CPC group, 867) compared the mean intraoperative blood loss. There was a significant heterogeneity in the literature (*P* < 0.00001, *I*^2^ = 92%). Meta-analysis was performed using random-effect model, and the result showed that intraoperative blood loss in the CPC group was significantly greater than that of in Zero-p group (WMD = − 13.30, 95% CI (− 18.83, − 7.78), *P* < 0.00001). The corresponding forest plot was shown in Fig. 3.

#### Length of stay (LOS)

**Fig. 4:**
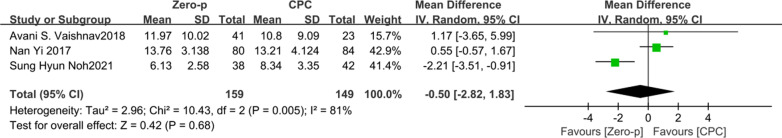
Meta-analysis of Zero-p group versus CPC group in length of stay

Three studies [8, 17, 28] consisting of 308 patients (Zero-p group, 159; CPC group, 149) compared the LOS. There was a significant heterogeneity in the literature (*P* = 0.005, *I*^2^ = 81%). Meta-analysis was performed using random-effect model, and the result showed that there was no significant difference in LOS between the Zero-p and CPC group (WMD = − 0.50, 95% CI (− 2.82, 1.83), *P* = 0.68). The corresponding forest plot was shown in Fig. 4.

#### NDI score

**Fig. 5:**
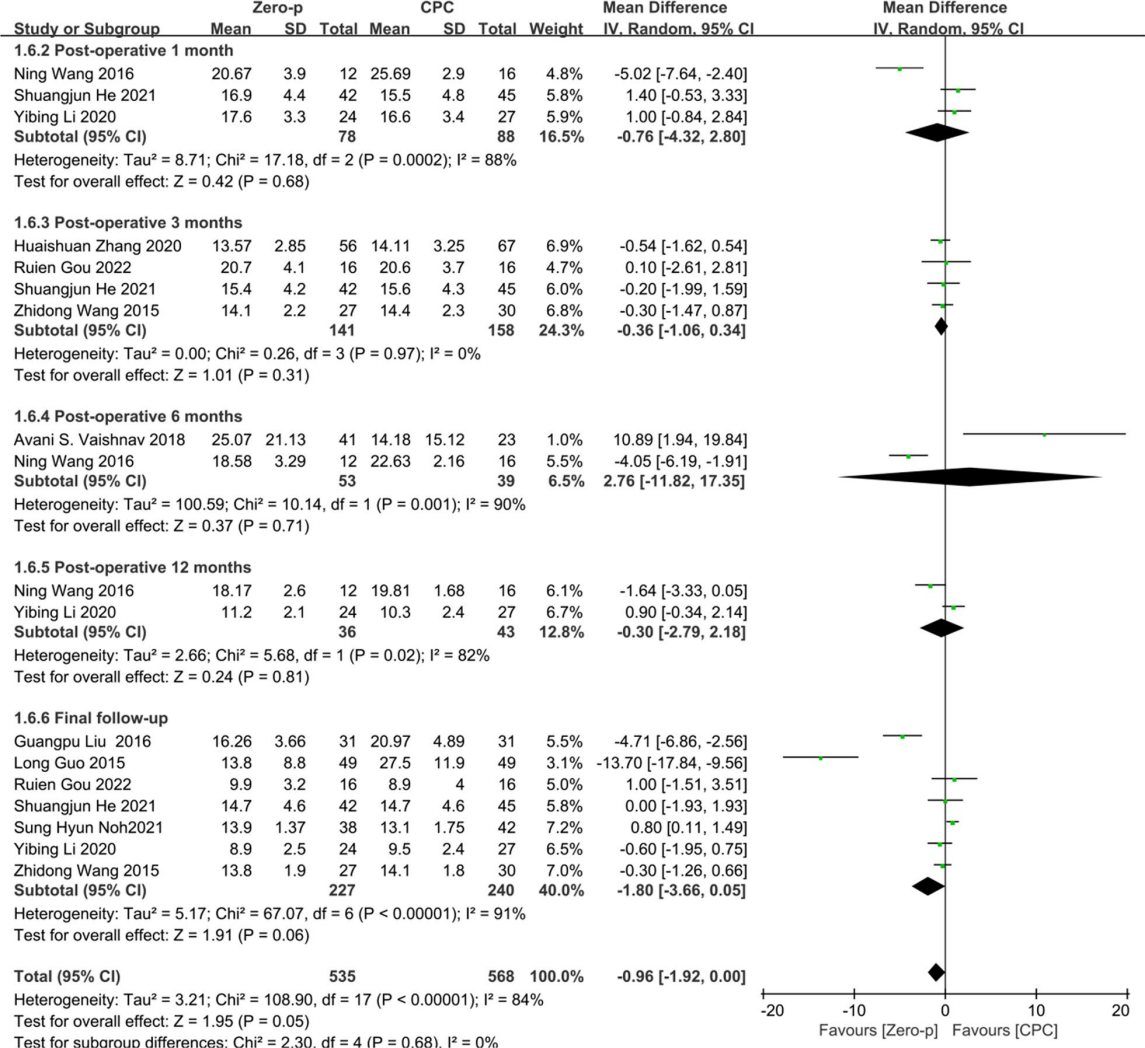
Meta-analysis of Zero-p group versus CPC group in NDI score

10 studies [8, 10, 16, 17, 23, 25, 26, 29, 30, 36] consisting of 682 patients (Zero-p group, 336; CPC group, 346) compared the NDI score. Three studies [16, 25, 29] reported the NDI score at postoperative 1 month. Four studies [10, 16, 23, 36] reported at postoperative 3 months. Two studies [8, 29] reported at postoperative 6 months. Two studies [25, 29] reported at postoperative 12 months. Seven studies [10, 16, 17, 23, 25, 26, 30] reported at final follow-up. No statistical difference was found preoperative period between the two groups (*P* = 0.65), (Additional file 2). There was a significant heterogeneity in the literature (*P* < 0.00001, *I*^2^ = 84%). Meta-analysis was performed using random-effect model and the results of subgroup analysis showed that there was no significant difference in NDI score between the Zero-p and CPC group after postoperative 1 month (WMD = − 0.76, 95% CI (− 4.32, 2.80), *P* = 0.68), postoperative 3 months (WMD = − 0.36, 95% CI (− 1.06, 0.34), *P* = 0.31), postoperative 6 months (WMD = 2.76, 95% CI (− 11.82, 17.35), *P* = 0.71), postoperative 12 months (WMD = − 0.30, 95% CI (− 2.79, 2.18), *P* = 0.81), and final follow-up (WMD = − 1.80, 95% CI (− 3.66, 0.05), *P* = 0.06). The corresponding forest plot was shown in Fig. 5.

#### JOA score

**Fig. 6:**
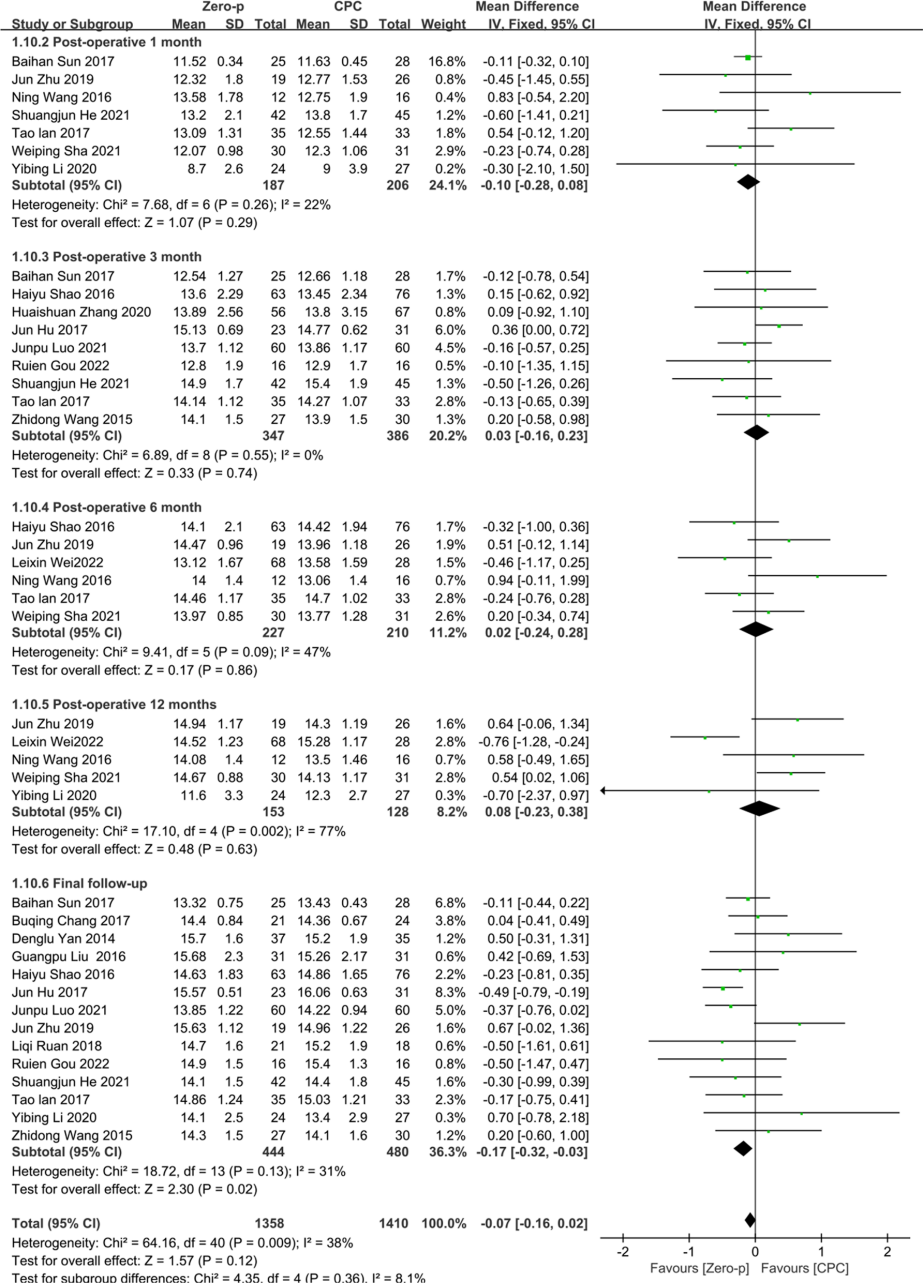
Meta-analysis of Zero-p group versus CPC group in JOA score

18 studies [7, 9–11, 16, 23–27, 29, 31–37] consisting of 1232 patients (Zero-p group, 610; CPC group, 622) compared the JOA score. Seven studies [7, 16, 25, 29, 31, 33, 35] reported the JOA score at postoperative 1 month. Nine studies [7, 10, 16, 23, 27, 31, 32, 36, 37] reported at postoperative 3 months. Five studies [7, 11, 27, 29, 33, 35] reported at postoperative 6 months. Five studies [11, 25, 29, 33, 35] reported at postoperative 12 months. 15 studies [7, 9, 10, 16, 23–27, 31, 32, 34, 35, 37] reported at final follow-up. No significant statistical difference was found in preoperative JOA score between the two groups (*P* = 0.08), (Additional file 3). There was no significant heterogeneity in the literature (*P* = 0.0009, *I*^2^ = 38%). Meta-analysis was performed using fixed-effect model, and the results of subgroup analysis showed that there was no significant difference in JOA score between the Zero-p and CPC group after postoperative 1 month (WMD = − 0.10, 95% CI (− 0.28, 0.08), *P* = 0.29), postoperative 3 months (WMD = 0.03, 95% CI (− 0.16, 0.23), *P* = 0.74), postoperative 6 months (WMD = 0.02, 95% CI (− 0.24, 0.28), *P* = 0.86), postoperative 12 months (WMD = 0.08, 95% CI (− 0.23, 0.38), *P* = 0.63), and final follow-up (WMD = − 0.17, 95% CI (− 0.32, − 0.03), *P* = 0.02). The corresponding forest plot was shown in Fig. 6.

### VAS

**Fig. 7:**
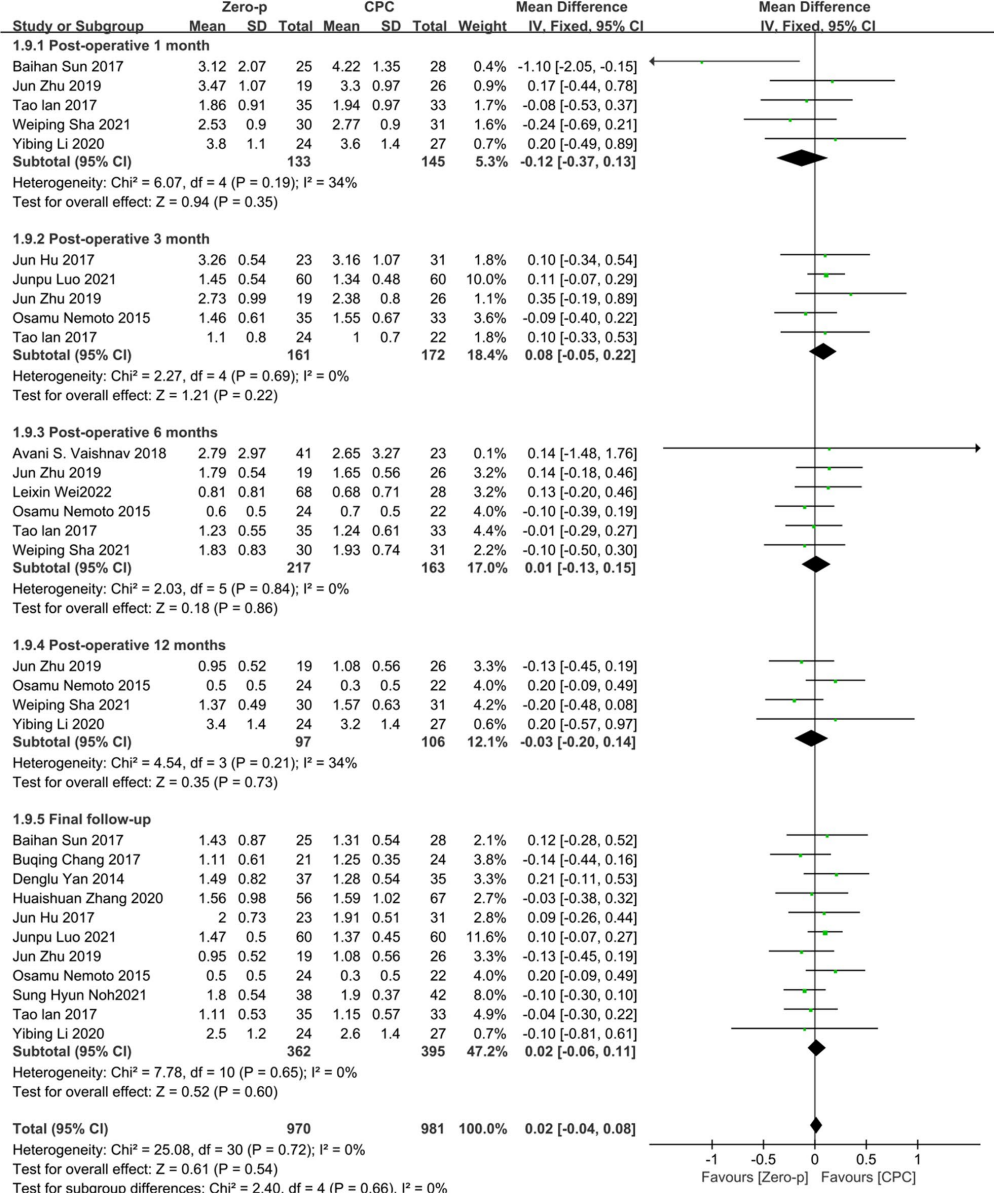
Meta-analysis of Zero-p group versus CPC group in VAS

14 studies [7–9, 11, 17, 20, 24, 25, 31–33, 35–37] consisting of 978 patients (Zero-p group, 501; CPC group, 477) compared the VAS. Five studies [7, 25, 31, 33, 35] reported the VAS at postoperative 1 month. Five studies [7, 20, 32, 35, 37] reported at postoperative 3 months. Six studies [7, 8, 11, 20, 33, 35] reported at postoperative 6 months. Four studies [20, 25, 33, 35] reported at postoperative 12 months. 11 studies [7, 9, 17, 20, 24, 25, 31, 32, 35–37] reported at final follow-up. No statistical difference was found in preoperative VAS between the two groups (*P* = 0.67), (Additional file 4). There was no significant heterogeneity in the literature (*P* = 0.72, *I*^2^ = 0%). Meta-analysis was performed using fixed-effect model, and the results of subgroup analysis showed that there was no significant difference in VAS between the Zero-p and CPC group after postoperative 1 month (WMD = − 0.12, 95% CI (− 0.37, 0.13), *P* = 0.35), postoperative 3 months (WMD = 0.08, 95% CI (− 0.05, 0.22), *P* = 0.22), postoperative 6 months (WMD = 0.01, 95% CI (− 0.13, 0.15), *P* = 0.86), postoperative 12 months (WMD = − 0.03, 95% CI (− 0.20, 0.14), *P* = 0.73), final follow-up (WMD = 0.02, 95% CI (− 0.06, 0.11), *P* = 0.60). The corresponding forest plot was shown in Fig. 7.

### Prevertebral soft tissue thickness (PSTT)

**Fig. 8:**
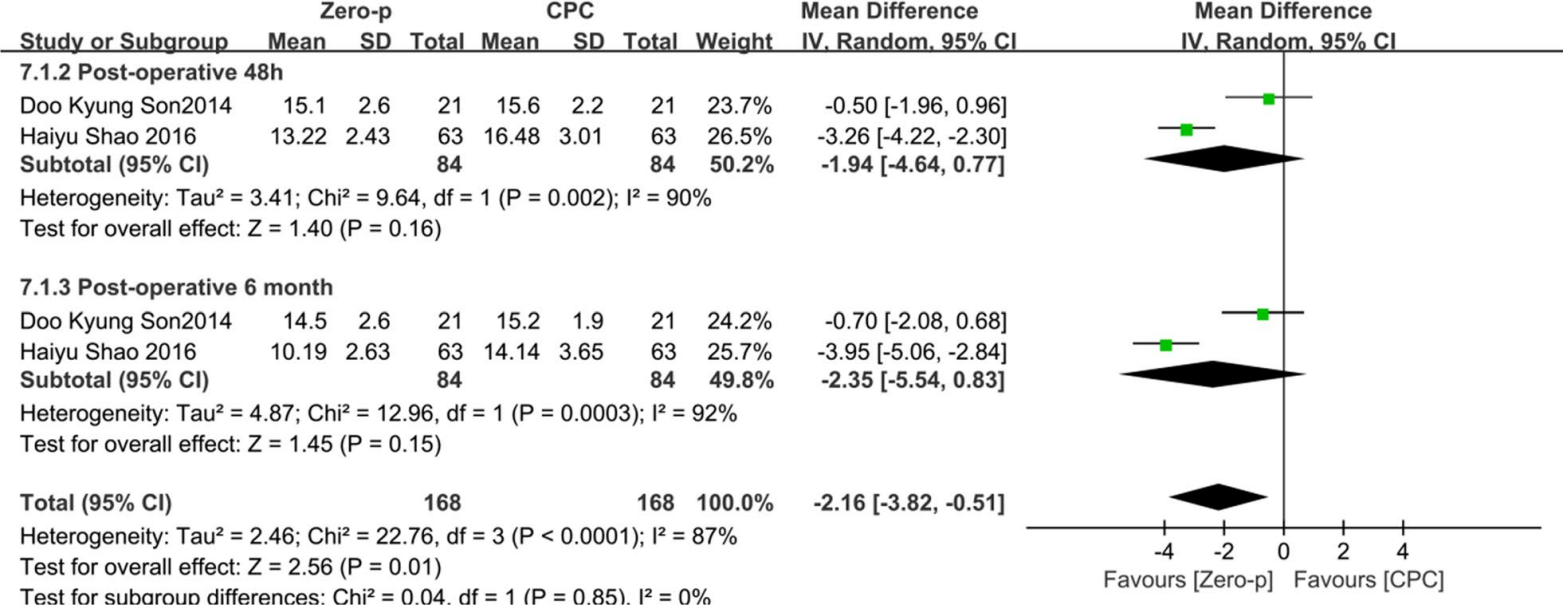
Meta-analysis of Zero-p group versus CPC group in PSTT

Two studies [19, 27] consisting of 168 patients (Zero-p group, 84; CPC group, 84) compared the PSTT. Two studies [19, 27] reported the PSTT at postoperative 48 h. Two studies [19, 27] reported at postoperative 6 months. No statistical difference was found preoperative period between the two groups (*P* = 0.90), (Additional file 5). There was significant heterogeneity in the literature (*P* < 0.0001, *I*^2^ = 87%). Meta-analysis was performed using random-effect model, and the results of subgroup analysis showed that there was no significant difference in PSTT between the Zero-p and CPC group after postoperative 24 h (WMD = − 1.94, 95% CI (− 4.64, 0.77), *P* = 0.16), and postoperative 6 months (WMD = − 2.35, 95% CI (− 5.54, 0.83), *P* = 0.15). The corresponding forest plot shown in Fig. 8.

### 36-Item Short Form Survey (SF-36)

**Fig. 9:**
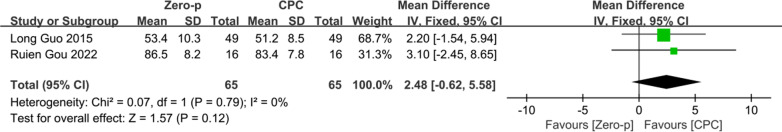
Meta-analysis of Zero-p group versus CPC group in SF-36

Two studies [10, 30] consisting of 130 patients (Zero-p group, 65; CPC group, 65) compared the SF-36. Two studies [10, 30] reported the SF-36 at the final follow-up. There was no significant heterogeneity in the literature (*P* = 0.79, *I*^2^ = 0%). Meta-analysis was performed using fixed-effect model, and the result showed that there was no significant difference in SF-36 between the Zero-p and CPC group at final follow-up (WMD = 2.48, 95% CI (− 0.62, 5.58), *P* = 0.12). The corresponding forest plot was shown in Fig. 9.

### Segmental Cobb angle

**Fig. 10:**
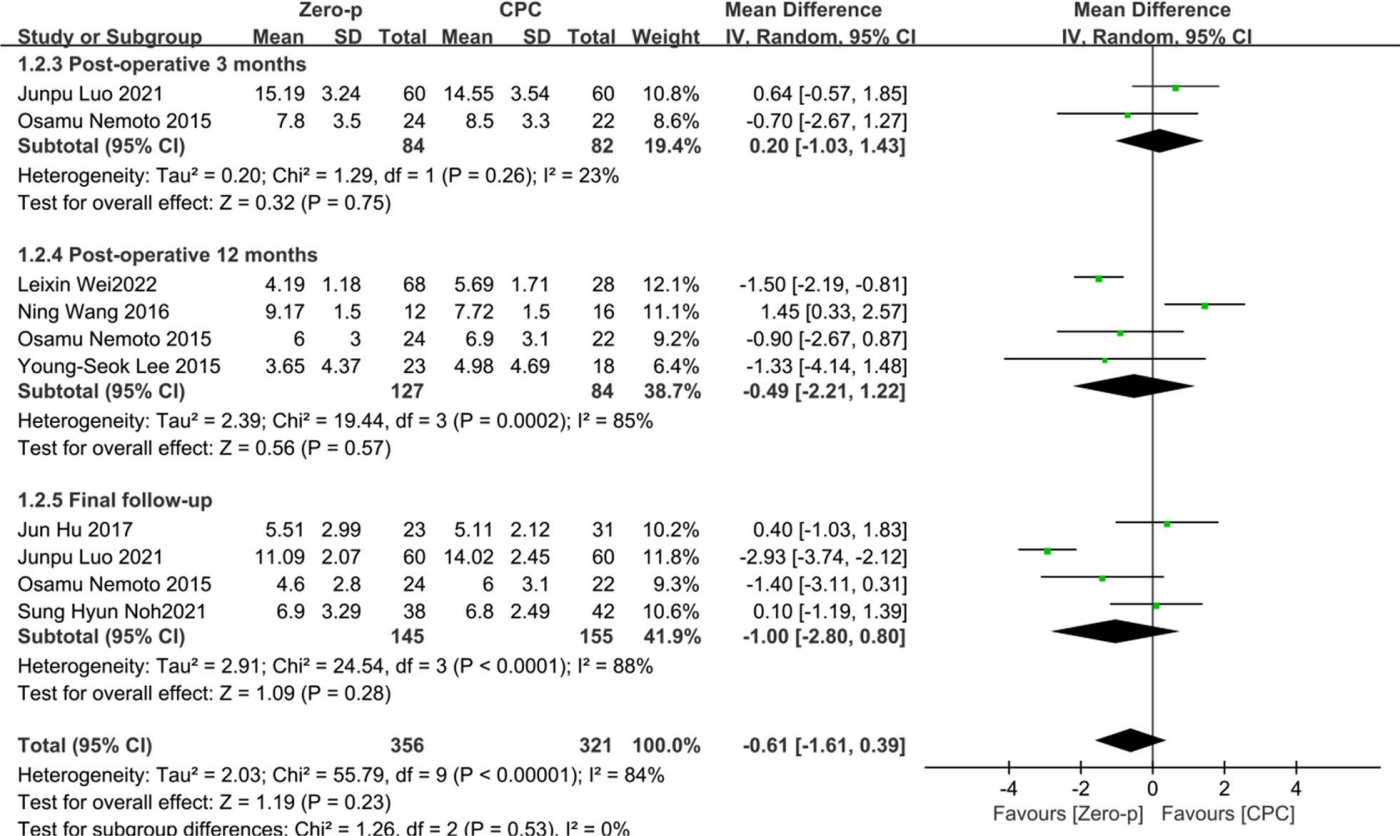
Meta-analysis of Zero-p group versus CPC group in postoperative segmental Cobb angle

Seven studies [11, 17, 18, 20, 29, 32, 37] consisting of 465 patients (Zero-p group, 248; CPC group, 217) compared the postoperative segmental Cobb angle. Two literature [20, 37] reported at postoperative 3 months. Four literature [11, 18, 20, 29] reported at postoperative 12 months. Four literature [17, 20, 32, 37] reported at final follow-up. There was significant heterogeneity in the literature (*P* < 0.00001, *I*^2^ = 84%). Meta-analysis was performed using random-effect model and the results of subgroup analysis showed that there was no significant difference in segmental Cobb angle between the Zero-p and CPC group after postoperative 3 month (WMD = 0.20, 95% CI (− 1.03, 1.43), *P* = 0.75), postoperative 6 months (WMD = − 0.49, 95% CI (− 2.21, 1.22), *P* = 0.57), and final follow-up (WMD = − 1.00, 95% CI (− 2.80, 0.80), *P* = 0.28). The corresponding forest plot was shown in Fig. 10.

### Cervical Cobb angle

**Fig. 11:**
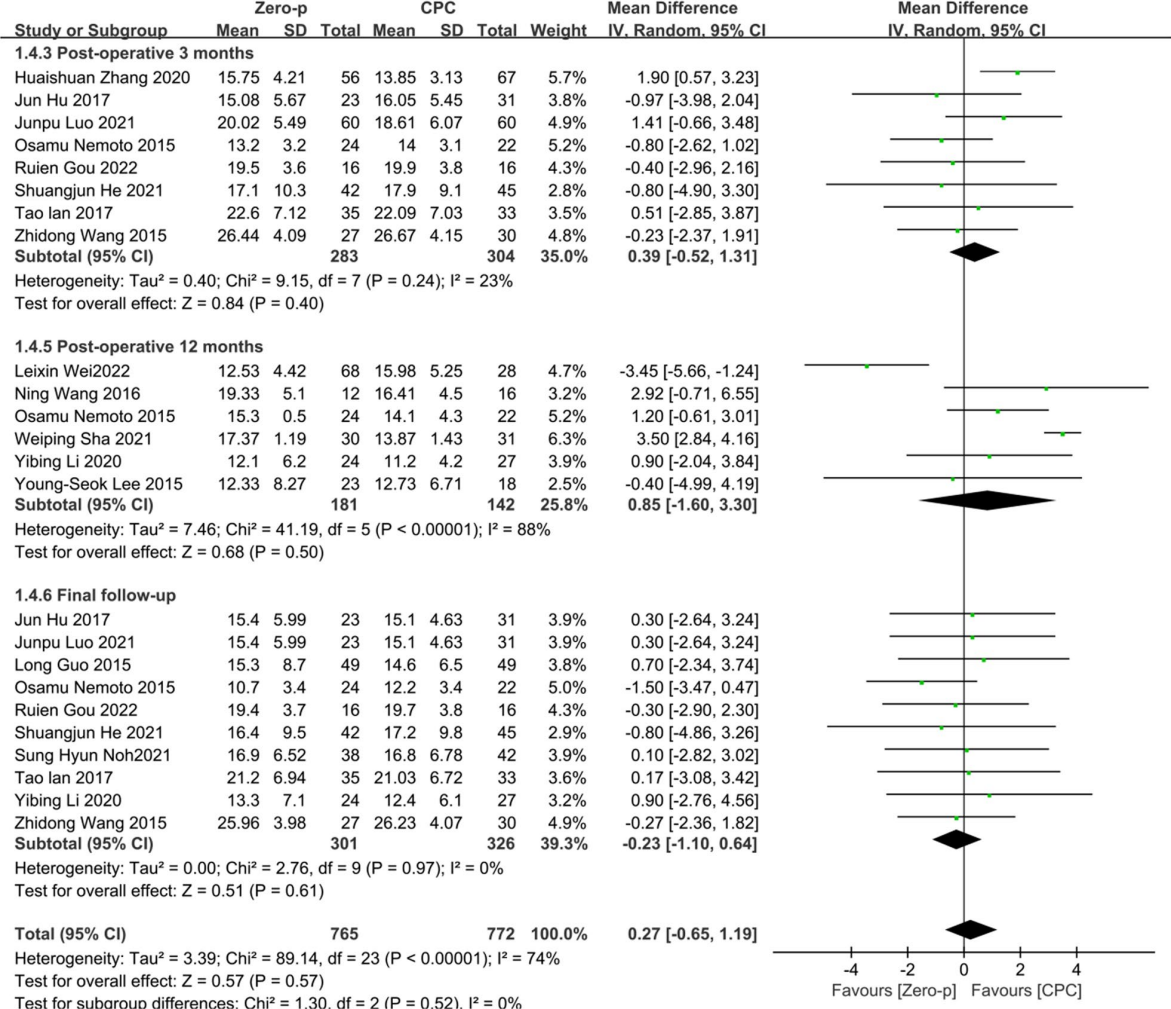
Meta-analysis of Zero-p group versus CPC group in postoperative cervical Cobb angle

15 studies [7, 10, 11, 16–18, 20, 23, 25, 29, 30, 32, 33, 36, 37] consisting of 1042 patients (Zero-p group, 527; CPC group, 515) compared the postoperative cervical Cobb angle. Eight literature [7, 16, 20, 23, 30, 32, 36, 37] reported at postoperative 3 months. Six literature [11, 18, 20, 25, 29, 33] reported at postoperative 12 months. 10 literature [7, 10, 16, 17, 20, 23, 25, 30, 31, 37] reported at final follow-up. There was significant heterogeneity in the literature (*P* < 0.00001, *I*^2^ = 74%). Meta-analysis was performed using random-effect model, and the results of subgroup analysis showed that there was no significant difference in cervical Cobb angle between the Zero-p and CPC group after postoperative 3 month (WMD = 0.39, 95% CI (− 0.52, 1.31), *P* = 0.40), postoperative 12 months (WMD = 0.85, 95% CI (− 1.60, 3.30), *P* = 0.50), and final follow-up (WMD = − 0.23, 95% CI (− 1.10, 0.64), *P* = 0.61). The corresponding forest plot was shown in Fig. 11.

#### Fusion rate

**Fig. 12:**
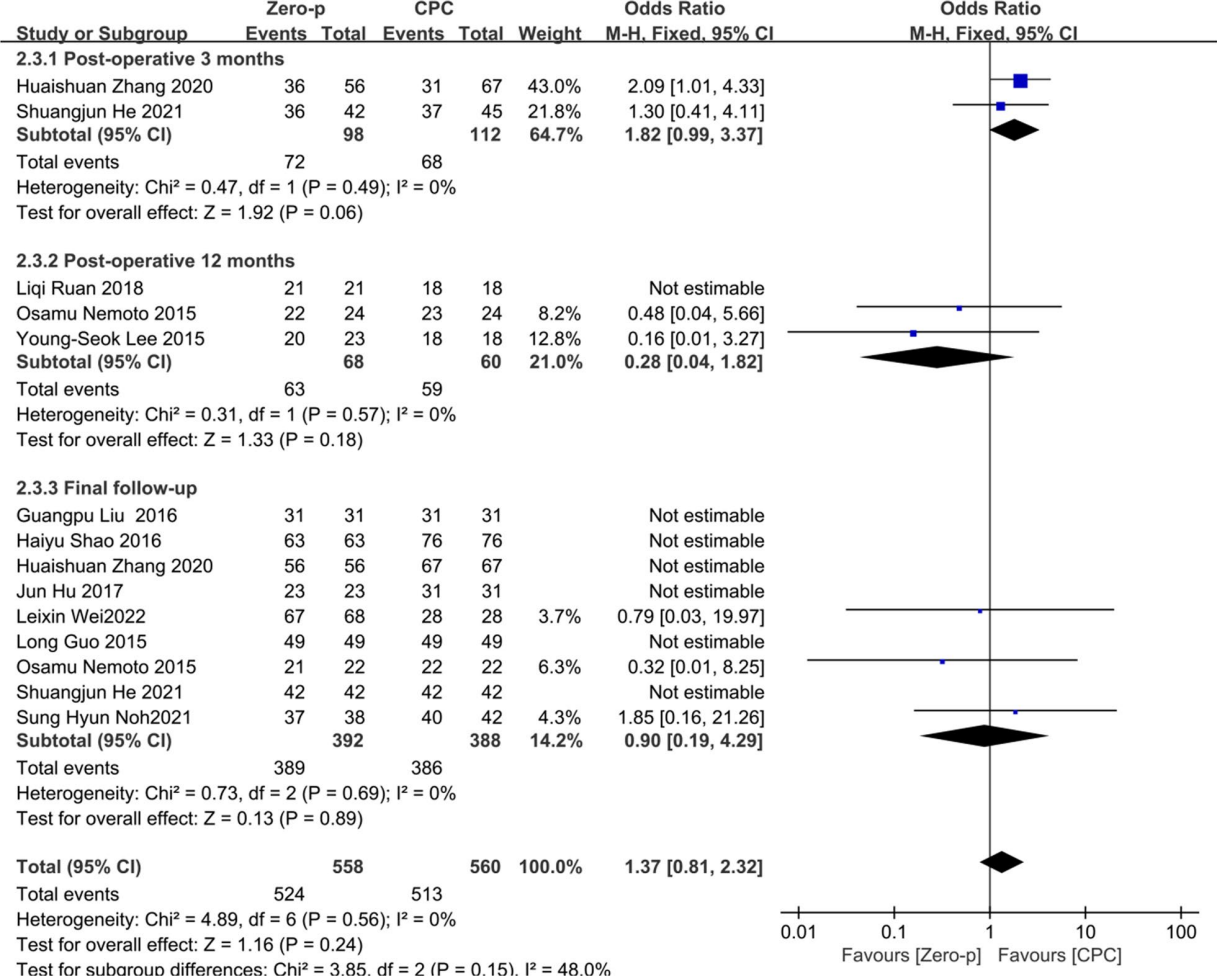
Meta-analysis of Zero-p group versus CPC group in fusion rate

10 studies [12, 16–18, 20, 26, 27, 30, 32, 34, 36] consisting of 819 patients (Zero-p group, 413; CPC group, 406) compared the fusion rate. Two studies [16, 36] reported the fusion rate at postoperative 3 months. Three studies [18, 20, 34] reported at postoperative 12 months. Nine studies [12, 16, 17, 20, 26, 27, 30, 32, 36] reported at final follow-up. There was no significant heterogeneity in the literature (*P* = 0.56, *I*^2^ = 0%). Meta-analysis was performed using fixed-effect model, and the results of subgroup analysis showed that there was no significant difference in fusion rate between the Zero-p and CPC group after postoperative 3 months (OR= 1.82, 95% CI (0.99, 3.37), *P* = 0.06), postoperative 12 months (OR= 0.28, 95% CI (0.04, 1.82), *P* = 0.18), and final follow-up (OR= 0.90, 95% CI (0.19, 4.29), *P* = 0.89). The corresponding forest plot was shown in Fig. 12.

## Complications

### Adjacent segment degeneration (ASD)

**Fig. 13:**
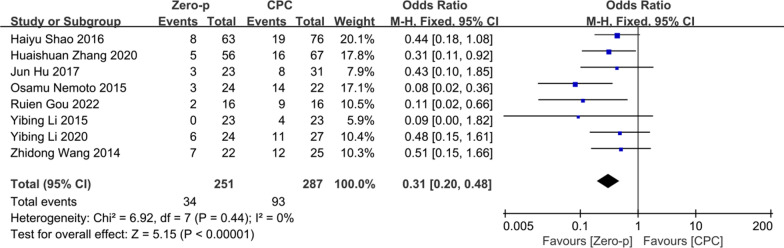
Meta-analysis of Zero-p group versus CPC group in ASD

Eight studies [10, 20–22, 25, 27, 32, 36] consisting of 538 patients (Zero-p group, 251; CPC group, 287) compared the ASD. There was no significant heterogeneity in the literature (*P* = 0.44, *I*^2^ = 0%). Meta-analysis was performed using fixed-effect model, and the result showed that there was a higher risk of ASD in the CPC group (OR= 0.31, 95% CI (0.20, 0.48), *P* < 0.0001). The corresponding forest plot was shown in Fig. 13.

#### Subsidence

**Fig. 14:**
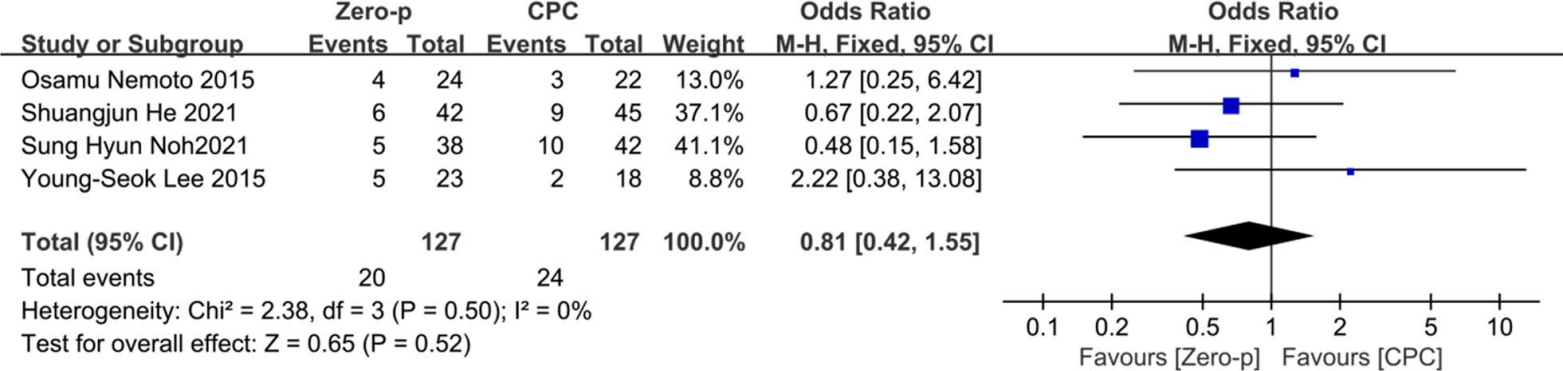
Meta-analysis of Zero-p group versus CPC group in cage subsidence

Four studies [16–18, 20] consisting of 254 patients (Zero-p group, 127; CPC group, 127) compared the cage subsidence. There was no significant heterogeneity in the literature (*P* = 0.50, *I*^2^ = 0%). Meta-analysis was performed using fixed-effect model, and the result showed there was no significant difference in subsidence between the Zero-p and CPC group (OR= 0.81, 95% CI (0.42, 1.55), *P* = 0.52). The corresponding forest plot was shown in Fig. 14.

#### Dysphagia

**Fig. 15:**
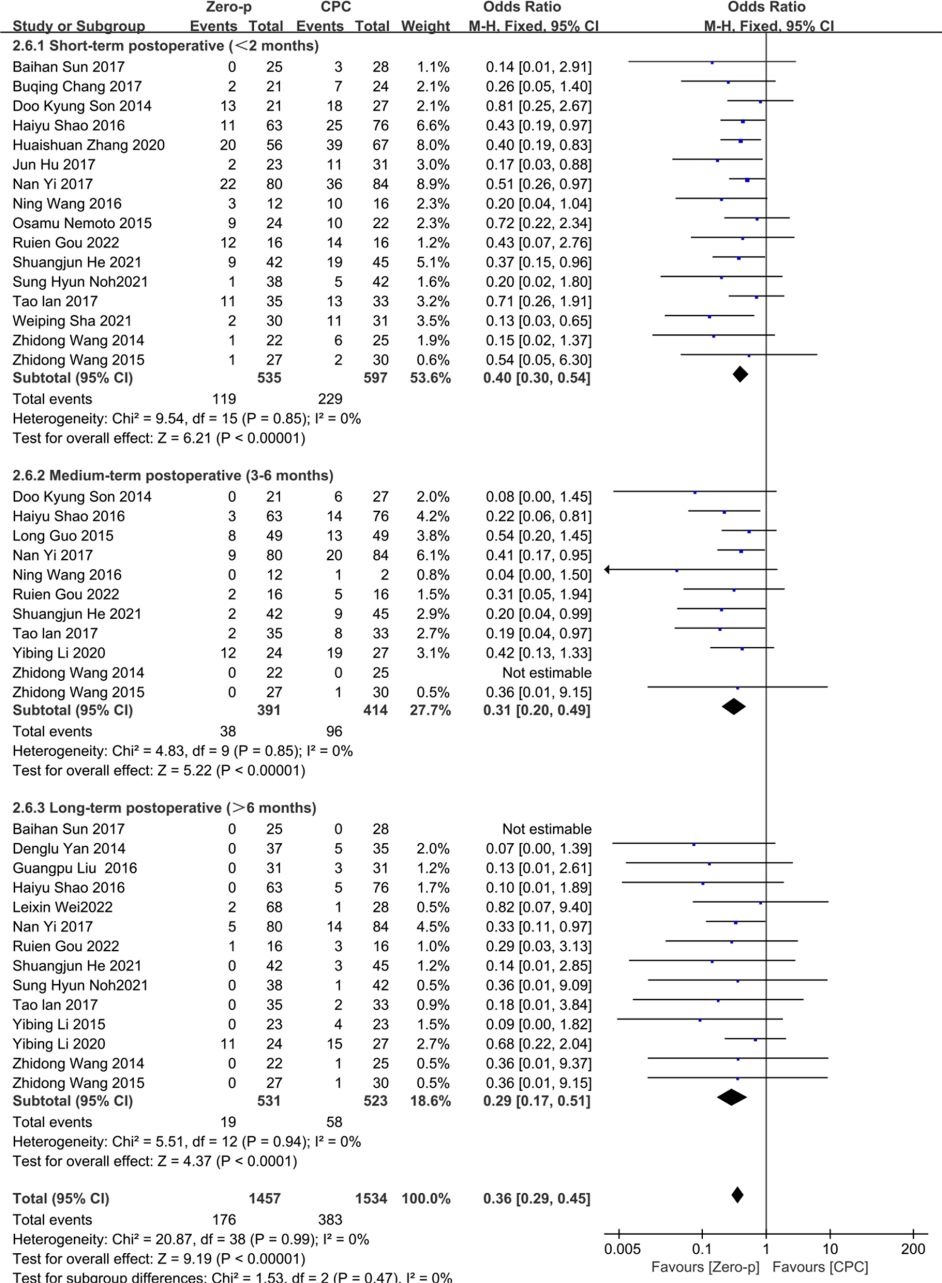
Meta-analysis of Zero-p group versus CPC group in postoperative dysphagia

22 studies [7, 9–11, 16, 17, 19–33, 36] consisting of 1557 patients (Zero-p group, 767; CPC group, 790) compared the incidence of postoperative dysphagia. 16 studies [7, 9, 10, 16, 17, 19–21, 23, 27–29, 31–33, 36] reported the short-term (< 2 months), 11 studies [7, 10, 16, 19, 21, 23, 25, 27–30] reported medium-term (3–6 months), and 14 studies [7, 10, 11, 16, 17, 21–28, 31] reported long-term (> 6 months) dysphagia. There was no significant heterogeneity in the literature (*P* = 0.99, *I*^2^ = 0%). Meta-analysis was performed using fixed-effect model, and the results of subgroup analysis showed that the CPC group had a higher risk of dysphagia in short term (OR= 0.40, 95% CI (0.30, 0.54), *P* < 0.00001), medium term (OR= 0.31, 95% CI (0.20, 0.49), *P* < 0.00001), and long term (OR= 0.29, 95% CI (0.17, 0.51), *P* < 0.0001). The corresponding forest plot analysis is shown in Fig. 15.

### Implant failure

**Fig. 16:**
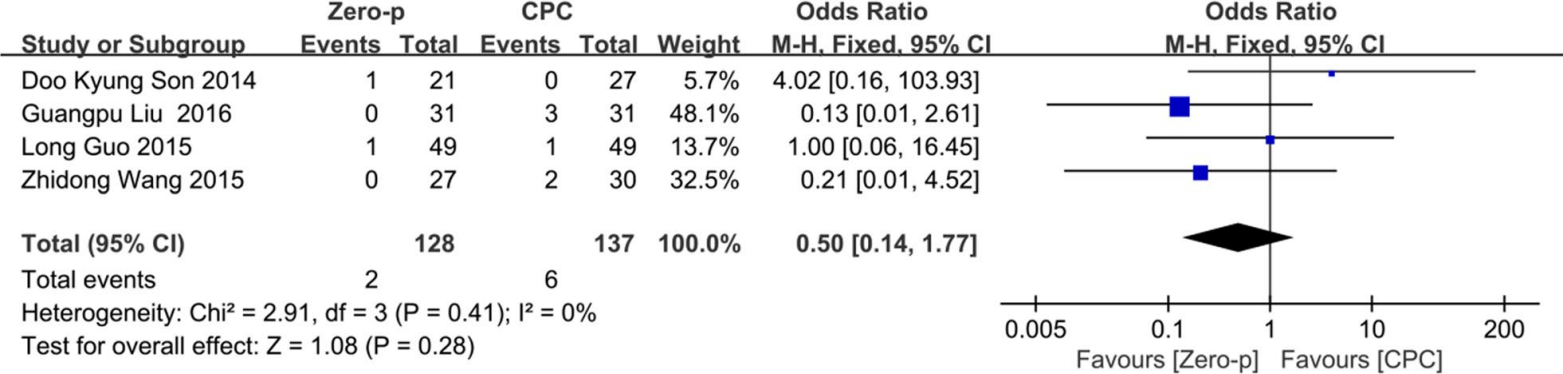
Meta-analysis of Zero-p group versus CPC group in postoperative implant failure

Four studies [19, 21, 26, 30] consisting of 265 patients (Zero-p group, 128; CPC group, 137) compared the incidence of implant failure. There was no significant heterogeneity in the literature (*P* = 0.41, *I*^2^ = 0%). Meta-analysis was performed using fixed-effect model, and the result showed there was no significant difference in the incidence of implant failure between the Zero-p and CPC group (OR= 0.50, 95% CI (0.14, 1.77), *P* = 0.28). The corresponding forest plot was shown in Fig. 16.

### Hoarseness

**Fig. 17:**
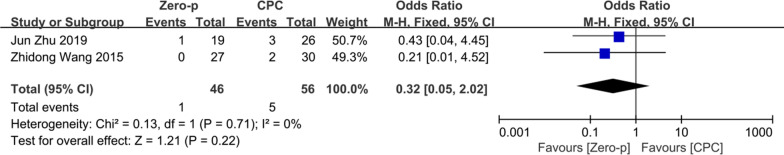
Meta-analysis of Zero-p group versus CPC group in postoperative hoarseness

Two studies [23, 35] consisting of 102 patients (Zero-p group, 46; CPC group, 56) compared the incidence of postoperative hoarseness. There was no significant heterogeneity in the literature (*P* = 0.71, *I*^2^ = 0%). Meta-analysis was performed using fixed-effect model, and the result showed there was no significant difference in the incidence of hoarseness between the Zero-p and CPC group (OR= 0.32, 95% CI (0.05, 2.02), *P* = 0.22). The corresponding forest plot was shown in Fig. 17.

### Publication bias and sensitivity analysis

**Fig. 18:**
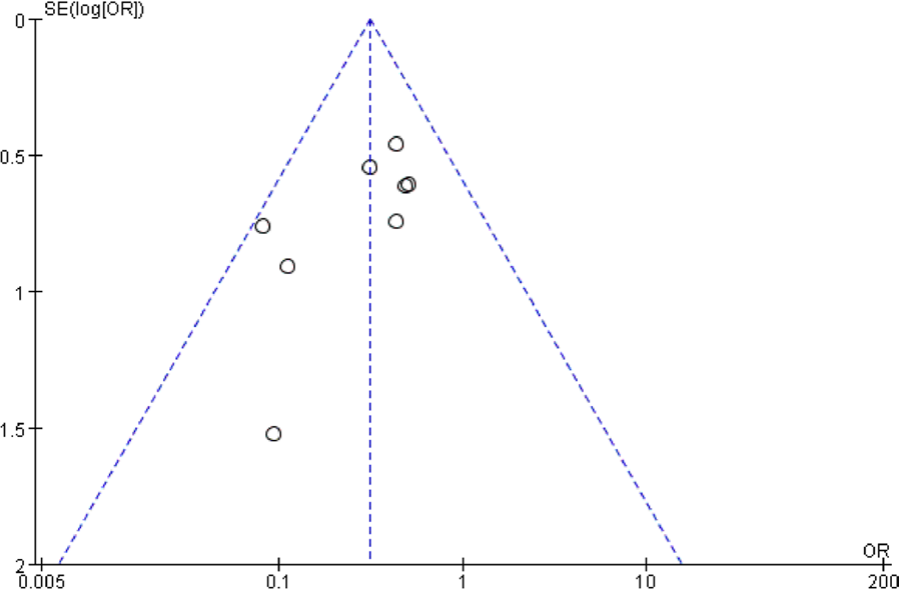
Funnel plot for publication bias of ASD at final follow-up

**Fig. 19:**
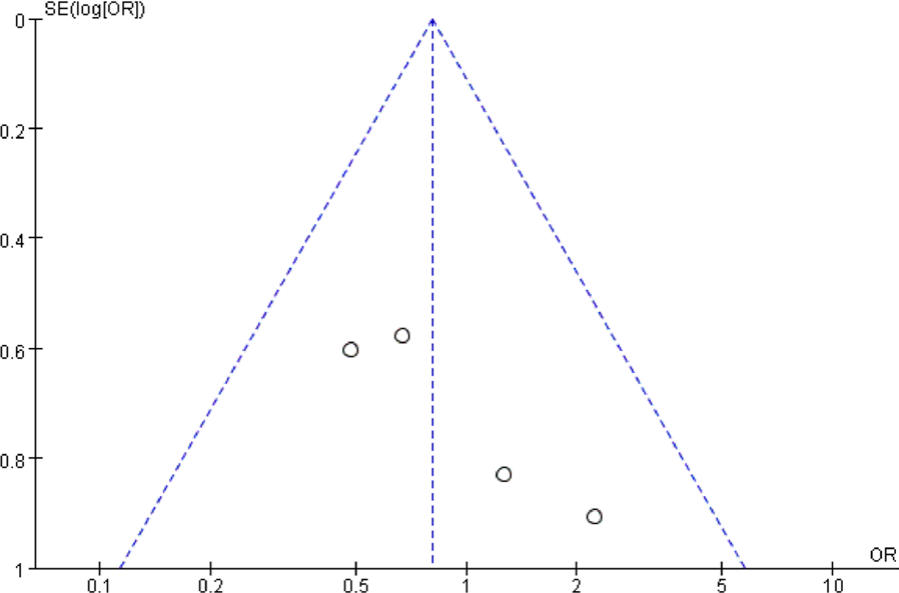
Funnel plot for publication bias of cage subsidence at final follow-up

**Fig. 20:**
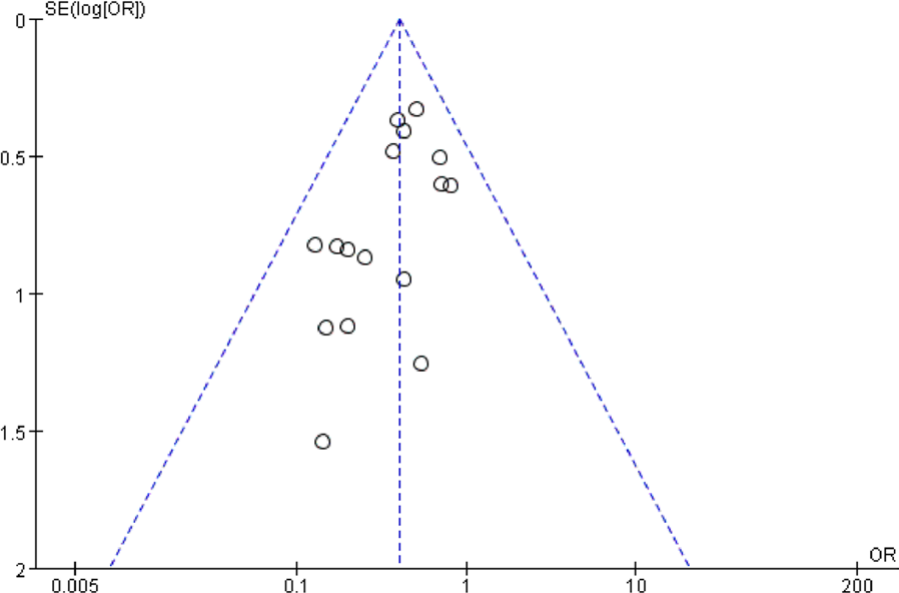
Funnel plot for publication bias of dysphagia at postoperative short-term

**Fig. 21:**
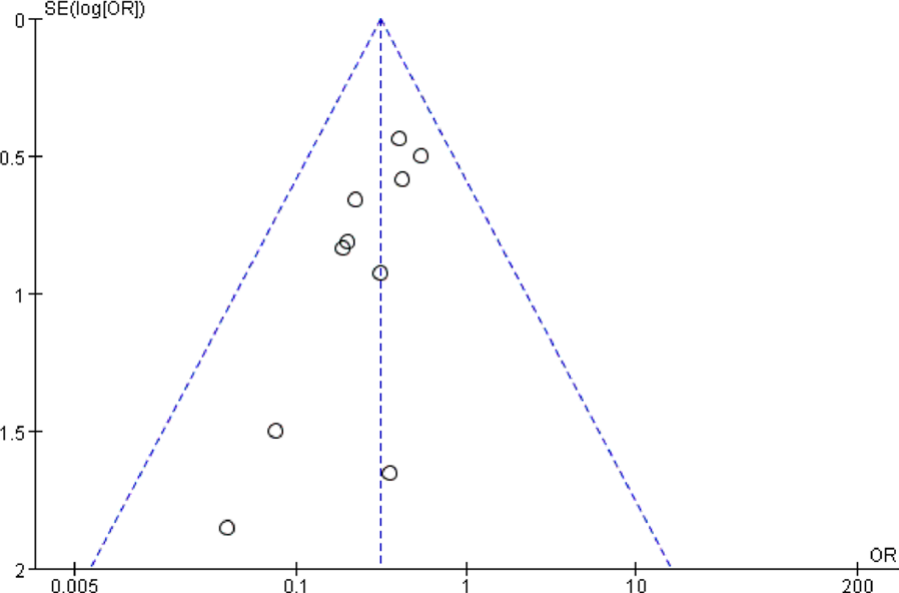
Funnel plot for publication bias of dysphagia at postoperative medium-term

**Fig. 22:**
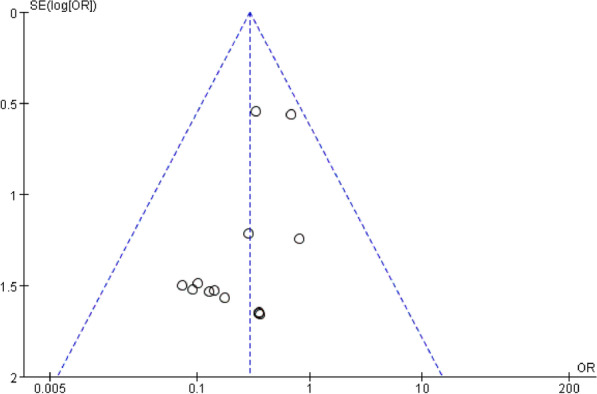
Funnel plot for publication bias of dysphagia at postoperative long-term

Funnel plots of the fusion rate, ASD, subsidence, dysphagia, and implant failure were shown in Fig. 18, 19, respectively. Funnel plots of the incidence of dysphagia at postoperative short term, medium term, and long term are shown in Fig. 20, 21, 22, respectively. All studies were within the 95% CI, indicating less publication bias. Sensitivity analysis by reanalyzing the data after sequential omission of individual studies revealed no significant changes.

## Discussion

DCS is a common cervical disease in adults, resulting in neck pain and decreased muscle strength of the extremities, seriously affecting the quality of life. After ineffective non-surgical treatment, patients with symptomatic DCS were often need surgical treatment. ACDF is a mainstay for the treatment of DCS [3, 38]. With the development of spinal internal fixation, CPC has been widely used in ACDF, which can address the needs of complete decompression, restoration of cervical physiological curvature, and improvement of stability and fusion rate. However, CPC has the risk of several defects such as dysphagia and tracheal injury. In addition, anterior irregularity of the vertebral body including osteophyte and mild spondylolisthesis in front of the vertebral body caused by degeneration of the cervical spine is often causing the postoperative loosening of the anterior cervical titanium plate and increasing the risk of ASD [5, 6, 39]. Zero-p, in addition to the advantages of CPC, can effectively avoid the disadvantages of CPC [7–10]. Several studies have been conducted about the therapeutic effects of Zero-p and CPC in recent years, but the sample sizes were small and there were no multi-center studies with large samples, leading to inconsistent conclusions. For this reason, this study is based on the differences in clinical efficacy between the two devices from an evidence-based medicine perspective to determine which device is more beneficial to the postoperative recovery of patients with DCS who underwent single-level ACDF.

In the aspect of operative time (*I*^2^ = 93%, *P* < 0.00001) and intraoperative blood loss (*I*^2^ = 92%, *P* < 0.00001), high heterogeneity among different studies may be related to the study type, sample size, and data statistics of the literature. Moreover, both of them were affected by the experience and surgical habits of the surgeon. Previously, Duan et al. [40] demonstrated that operative time between the Zero-p group and CPC group in one-level ACDF was not significant, but there was a significant reduction in intraoperative blood loss. In contrast, Nambiar et al. [41] demonstrated that Zero-p significantly reduced the operative time, but was inferior in significantly reducing the intraoperative blood loss. The shortening of operative time and intraoperative blood loss will help to reduce the occurrence of perioperative risks and complications. It is superior in the postoperative rehabilitation of patients to the CPC group. There is no significant difference in LOS between the two groups, while Nambiar et al. [41] did not discuss the LOS in the previous meta-analysis. There is no significant difference in SF-36 between the two groups. It indicates that the influence of two devices on physical and mental health was insignificant.

In this study, we found that there was no statistically significant difference in postoperative VAS and NDI scores in the Zero-p CPC group. The JOA score in Zero-p group was significantly higher at follow-up (WMD = − 0.17, 95% CI (− 0.32, − 0.03), *P* = 0.02). The results suggest that Zero-p can achieve the same clinical efficacy as CPC in single-level ACDF. Nambiar et al. [41] and Lu et al. [12] had similar results in a meta-analysis of Zero-p versus CPC in single-level and two-level ACDF, respectively.

In terms of radiological outcomes, the differences of the postoperative segmental and cervical Cobb angle between the two groups were not significant. This was consistent with the results of Nambiar et al. [41]. It indicates that Zero-p and CPC groups were equally effective in restoring cervical curvature in single-level ACDF. Perrini et al. [42] reported that CPC was more conducive to the recovery of cervical curvature during two-level ACDF. Dong et al. [43] revealed that CPC was significantly superior in maintaining the segmental Cobb angle. No articles with single-level ACDF were included in Yang et al. [44], and only two articles with single-level ACDF were included in Sun et al. [45], and the results revealed a significant increase in cervical lordosis in the CPC group, but the current meta-analysis revealed no significant difference in both segmental and cervical Cobb between Zero-p and CPC group, indicating that Zero-p has similar efficacy in maintaining the segmental curvature with CPC in single-level ACDF. Thus, we recommend using Zero-p in single-level ACDF, but not in multi-level ACDF.

In addition, the PSTT in the Zero-p group was thinner, attributing to the smaller surgical exposure, milder stimulus to the prevertebral soft tissue and esophagus, and preserving anatomical tissues. Both two sub-groups analyses were not significant, possibly due to the limited sample size resulting in low statistical power comparison. There was no significant difference in fusion rate between the two groups at 3 months, 12 months, and final follow-up (73.5% VS 60.7%; 92.6% VS 98.3%; 99.2% VS 99.5%). Zero-p was demonstrated to provide good postoperative stability in single-level ACDF, consistent with Duan et al. [40], Nambiar et al. [41], and Dong et al. [43]. Scholz et al. [46] demonstrated that both two devices provide the same biomechanical environment, leading to the similar fusion rates.

In terms of the postoperative complications in the two groups, the Zero-p group significantly reduced the incidence of ASD (13.5% VS 32.4%), which was not mentioned by Nambiar et al. [41]. Chuang et al. [5] demonstrated that the distance between the edge of plate and adjacent segment less than 5 mm was a risk factor for ASD. Zero-p, however, is far from the adjacent segment and reduces the incidence of ASD. Liu et al. [47] demonstrated that CPC had a significantly higher subsidence rate, but Nambiar et al. [41] demonstrated that it was similar. Previously reported subsidence rates of Zero-P were not accordant. The result of this study was consistent with Nambiar et al. [41]. It indicates that Zero-p does not increase the risk of subsidence in single-level ACDF. Kim et al. [48] demonstrated that the presence of subsidence was significantly associated with adverse clinical outcomes. The results of this study showed that the incidence of dysphagia in the Zero-p group was significantly lower than that in the CPC group in postoperative short term (< 2 months), medium term (3–6 months), and long term (> 6 months), (22.2% VS 38.4%, 9.71% VS 23.2%, 3.6% VS 11.1%). Therefore, the use of zero-p can significantly reduce the incidence of postoperative dysphagia. Fountas et al. [6] demonstrated that postoperative dysphagia may be related to prevertebral soft tissue edema and adhesion, postoperative hematoma, and esophageal injury. Accordingly, postoperative PSTT in the Zero-p group was thinner in this study (Fig. 8). The reason why intraoperative blood loss was significantly reduced in the Zero-p group was less damage to the soft tissues and blood vessels around the esophagus. In addition, this result may explain why the incidence of postoperative dysphagia is low in the Zero-p group. Lu et al. [12] reported that there was no significant difference between the stand-alone cage group and CPC group in contiguous two-level ACDF. Nambiar et al. [41] demonstrated an insignificant difference in postoperative dysphagia, but significant at the final follow-up. The reason why this was different from the results of our study may be due to the fewer included literature. There were significant differences between two groups in the early postoperative period, 3 months postoperative period, and the final follow-up in Lu et al. [49] and the postoperative period in Zhang et al. [50], but both studies included single-level and multi-level ACDF. Incidence of implant failure and hoarseness were not reported in the previous meta-analysis [12, 40, 41, 49, 50]. In this study, there was no significant difference in both incidence of implant failure and hoarseness between the two groups.

This study also has some limitations, such as not only including RCTs but also including retrospective studies. Different regions, populations, and ethnicities may also have some impact on the results. In addition, the lack of both surgical and hospitalization costs in the included literature resulted in the inability to comprehensively compare the advantages and disadvantages of the two devices. Further high-quality meta-analyses are still needed to validate the results of this study.

## Conclusions

In conclusion, Zero-p in single-level ACDF has significant advantages because it reduces the operative time, intraoperative blood loss, JOA score at follow-up, and the incidence of postoperative ASD and dysphagia. However, Zero-p and CPC have similar efficacy in terms of postoperative VAS, NDI, LOS, fusion rate, segmental Cobb angle, cervical Cobb angle, PSTT, SF-36, subsidence, implant failure, and hoarseness. The use of Zero-p in single-level ACDF was recommended.

## Supplementary Information

**Additional file 1**. (A) Meta-analysis of Zero-p group versus CCP group in age; (B) Meta-analysis of Zero-p group versus CCP group in BMD; (C) Meta-analysis of Zero-p group versus CCP group in BMI; (D) Meta-analysis of Zero-p group versus CCP group in follow-up time.

**Additional file 2**. Meta-analysis of Zero-p group versus CCP group in preoperative NDI score.

**Additional file 3**. Meta-analysis of Zero-p group versus CCP group in preoperative JOA score.

**Additional file 4**. Meta-analysis of Zero-p group versus CCP group in preoperative VAS score.

**Additional file 5**. Meta-analysis of Zero-p group versus CCP group in preoperative PSTT.

## Data Availability

The data used to support the findings of this study are included within the article.

## Abbreviations

ACDF: Anterior cervical discectomy and fusion
CPC: Cervical cage–plate construct
JOA: Japanese Orthopaedic Association
NDI: Neck Disability Index
VAS: Visual analog scale
PSTT: Prevertebral soft tissue thickness
SF-36: 36-Item Short Form Survey
ASD: Adjacent segment degeneration
