# Supplementary material for: Zero-profile implant versus conventional cage–plate construct in anterior cervical discectomy and fusion for the treatment of single-level degenerative cervical spondylosis: a systematic review and meta-analysis
Source: J Orthop Surg Res. 2022 Nov 24;17:506. doi: 10.1186/s13018-022-03387-9 (PMC9694547; doi:10.1186/s13018-022-03387-9)
Supplement: Supplementary file 1 — Additional file 1. (A) Meta-analysis of Zero-p group versus CCP group in age; (B) Meta-analysis of Zero-p group versus CCP group in BMD; (C) Meta-analysis of Zero-p group versus CCP group in BMI; (D) Meta-analysis of Zero-p group versus CCP group in follow-up time. [file 13018_2022_3387_MOESM1_ESM.pdf]

A

Study or Subgroup	Zero-p Mean	Zero-p SD	Zero-p Total	CPC Mean	CPC SD	CPC Total	Weight	Mean Difference IV, Fixed, 95% CI
Avani S. Vaishnav2018	48.58	10.72	41	46.37	8.4	23	2.6%	2.21 [-2.54, 6.96]
Baihan Sun 2017	53	10.26	25	53.57	10.66	28	1.9%	-0.57 [-6.21, 5.07]
Buqing Chang 2017	54.6	3.5	21	53.2	4.2	24	11.7%	1.40 [-0.85, 3.65]
Denglu Yan2014	63.55	7.12	37	64.28	8.76	35	4.3%	-0.73 [-4.43, 2.97]
Doo Kyung Son2014	55.4	9.7	21	50.2	10.9	27	1.7%	5.20 [-0.64, 11.04]
Guangpu Liu 2016	48.5	9.1	31	45.2	10.6	31	2.5%	3.30 [-1.62, 8.22]
Haiyu Shao 2016	47.6	6.4	63	50.3	8.2	76	10.1%	-2.70 [-5.13, -0.27]
Huaishuan Zhang 2020	45.2	13.9	56	48.7	13.2	67	2.6%	-3.50 [-8.32, 1.32]
Jun Hu2017	49.78	10.4	23	45.54	40.22	31	0.3%	4.24 [-10.54, 19.02]
Junpu Luo 2021	59.1	16.4	60	59.8	14.4	60	1.9%	-0.70 [-6.22, 4.82]
Jun Zhu 2019	55.42	8.03	19	59.15	8.04	26	2.6%	-3.73 [-8.48, 1.02]
Leixin Wei2022	48.7	7.3	68	47.2	6.7	28	6.5%	1.50 [-1.53, 4.53]
Liqi Ruan 2018	56.3	9.8	21	59.6	12.5	18	1.2%	-3.30 [-10.44, 3.84]
Long Guo 2015	43.1	16.9	49	43.3	17.7	49	1.3%	-0.20 [-7.05, 6.65]
Nan Yi 2017	52.12	5.893	80	51.95	6.267	84	17.1%	0.17 [-1.69, 2.03]
Osamu Nemoto2015	40.9	7.2	24	41.6	7	22	3.5%	-0.70 [-4.81, 3.41]
Ruien Gou 2022	48.5	6.7	16	52.4	7.2	16	2.6%	-3.90 [-8.72, 0.92]
Shuangjun He2021	62.59	8.21	42	61.15	7.52	45	5.4%	1.44 [-1.88, 4.76]
Sung Hyun Noh2021	51.9	10.21	38	52.6	8.61	42	3.4%	-0.70 [-4.86, 3.46]
Tao Lan2017	54.05	10.11	35	52.09	10.46	33	2.5%	1.96 [-2.93, 6.85]
Weiping Sha 2021	52.9	9.27	30	50.33	8.57	31	3.0%	2.57 [-1.91, 7.05]
Yibing Li2020	65.7	7.5	24	62.3	3.4	27	5.6%	3.40 [0.14, 6.66]
Young-Seok Lee2015	57.26	13.28	23	52.89	7.71	18	1.4%	4.37 [-2.12, 10.86]
Zhidong Wang2014	50.86	8.79	22	53.68	8.96	25	2.3%	-2.82 [-7.90, 2.26]
Zhidong Wang2015	51.6	11.3	27	54	8.5	30	2.2%	-2.40 [-7.64, 2.84]
**Total (95% CI)**			**896**			**896**	**100.0%**	**0.16 [-0.61, 0.93]**

Heterogeneity: Chi² = 31.67, df = 24 (P = 0.14); I² = 24%
Test for overall effect: Z = 0.42 (P = 0.68)

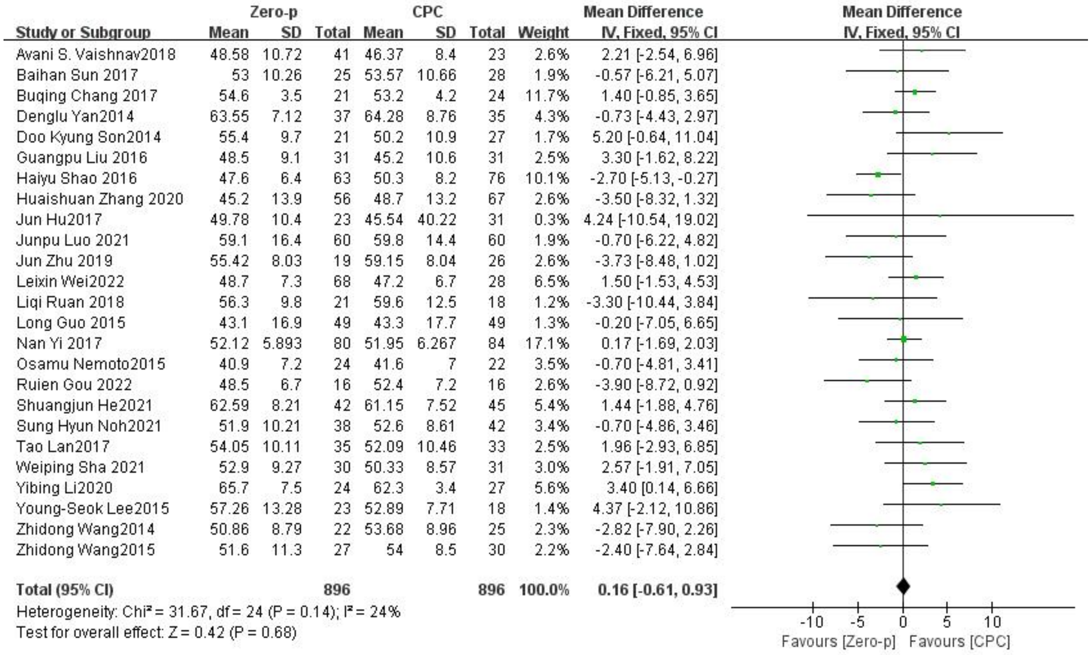

Supplementary material. 1 A Meta-analysis of Zero-p group versus CCP group in age

B

Study or Subgroup	Zero-p Mean	Zero-p SD	Zero-p Total	CPC Mean	CPC SD	CPC Total	Weight	Mean Difference IV, Fixed, 95% CI
Sung Hyun Noh2021	-0.75	0.47	38	-0.71	0.42	42	96.4%	-0.04 [-0.24, 0.16]
Young-Seok Lee2015	-0.74	1.83	23	-0.36	1.5	18	3.6%	-0.38 [-1.40, 0.64]
**Total (95% CI)**			**61**			**60**	**100.0%**	**-0.05 [-0.24, 0.14]**

Heterogeneity: Chi² = 0.41, df = 1 (P = 0.52); I² = 0%
Test for overall effect: Z = 0.53 (P = 0.60)

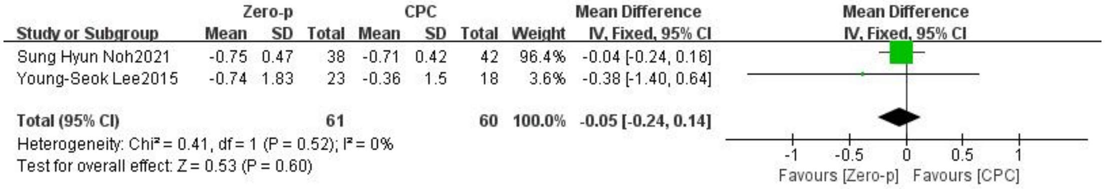

Supplementary material. 1 B Meta-analysis of Zero-p group versus CCP group in BMD

C

Study or Subgroup	Zero-p Mean	Zero-p SD	Zero-p Total	CPC Mean	CPC SD	CPC Total	Weight	Mean Difference IV, Fixed, 95% CI
Avani S. Vaishnav2018	30.45	5.12	41	28.94	5.13	23	8.4%	1.51 [-1.11, 4.13]
Leixin Wei2022	22.8	2.2	68	22.5	1.8	28	79.9%	0.30 [-0.55, 1.15]
Sung Hyun Noh2021	22.4	4.31	38	22.8	5.75	42	11.7%	-0.40 [-2.61, 1.81]
**Total (95% CI)**			**147**			**93**	**100.0%**	**0.32 [-0.44, 1.08]**

Heterogeneity: Chi² = 1.20, df = 2 (P = 0.55); I² = 0%
Test for overall effect: Z = 0.83 (P = 0.41)

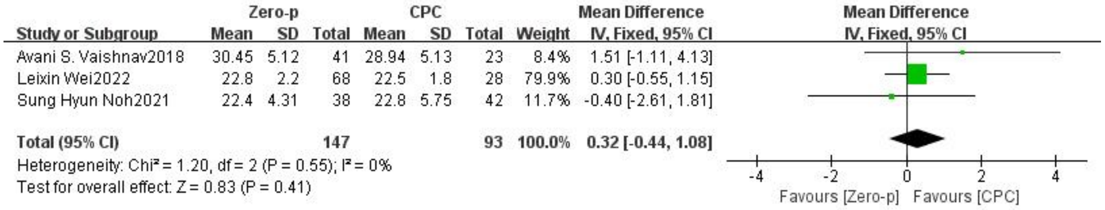

Supplementary material. 1 C Meta-analysis of Zero-p group versus CCP group in BMI

D

Study or Subgroup	Zero-p Mean	Zero-p SD	Zero-p Total	CPC Mean	CPC SD	CPC Total	Weight	Mean Difference IV, Random, 95% CI
Buqing Chang 2017	14	2	21	14	2	24	8.2%	0.00 [-1.17, 1.17]
Denglu Yan2014	15.32	2.13	37	14.26	2.35	35	8.5%	1.06 [0.02, 2.10]
Guangpu Liu 2016	15.52	1.93	31	16.1	2.33	31	8.5%	-0.58 [-1.65, 0.49]
Haiyu Shao 2016	23.6	4.5	63	25.2	4.8	76	7.3%	-1.60 [-3.15, -0.05]
Huaishuan Zhang 2020	21.46	4.51	56	21.46	4.51	67	7.1%	0.00 [-1.60, 1.60]
Jun Hu2017	15.7	2.4	23	15.7	2.4	31	7.9%	0.00 [-1.29, 1.29]
Leixin Wei2022	15.3	5.2	68	15.1	5.2	28	5.5%	0.20 [-2.09, 2.49]
Liqi Ruan 2018	13.3	1.9	21	14.9	1.7	18	8.3%	-1.60 [-2.73, -0.47]
Long Guo 2015	18.5	17.5	49	18.5	17.5	49	1.2%	0.00 [-6.93, 6.93]
Osamu Nemoto2015	24	0	24	24	0	22		Not estimable
Shuangjun He2021	22.6	3.3	42	27.1	3.5	45	7.6%	-4.50 [-5.93, -3.07]
Sung Hyun Noh2021	37.6	5.91	38	37.1	15.7	42	2.0%	0.50 [-4.61, 5.61]
Tao Lan2017	23.68	1.93	35	24.39	2	33	8.8%	-0.71 [-1.65, 0.23]
Weiping Sha 2021	13.5	1.5	30	13.5	1.5	31	9.2%	0.00 [-0.75, 0.75]
Yibing Li2013	24	0	23	24	0	23		Not estimable
Yibing Li2020	81	4.4	24	79	3.4	27	5.7%	2.00 [-0.18, 4.18]
Young-Seok Lee2015	12.57	2.09	23	28.89	20.24	18	0.7%	-16.32 [-25.71, -6.93]
Zhidong Wang2014	33.59	5.52	22	33.16	5.97	25	3.7%	0.43 [-2.86, 3.72]
**Total (95% CI)**			**630**			**625**	**100.0%**	**-0.57 [-1.37, 0.23]**

Heterogeneity: Tau² = 1.67; Chi² = 64.70, df = 15 (P < 0.00001); I² = 77%
Test for overall effect: Z = 1.39 (P = 0.16)

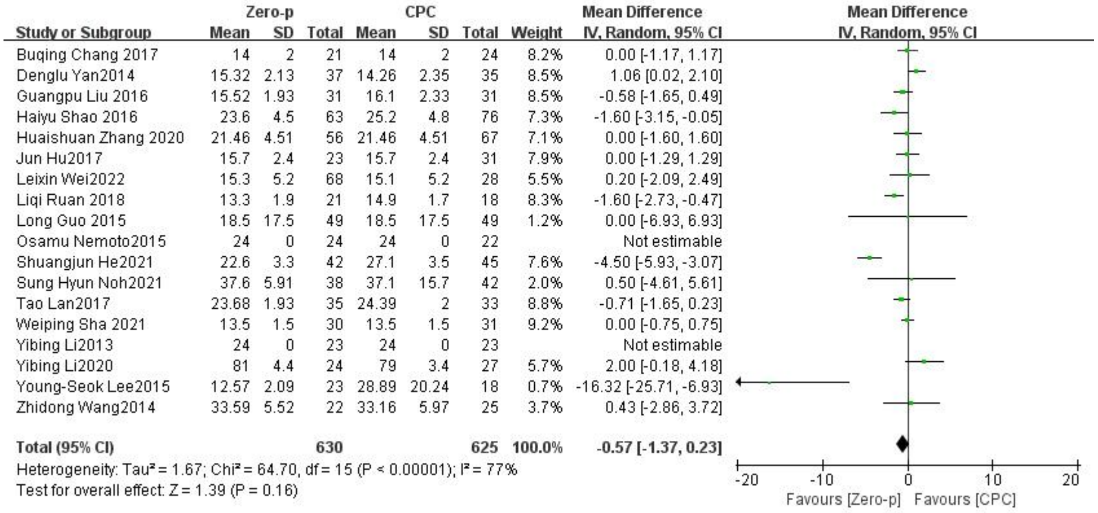

Supplementary material. 1 D Meta-analysis of Zero-p group versus CCP group in follow-up time
