# Supplementary material for: Zero-profile implant versus conventional cage–plate construct in anterior cervical discectomy and fusion for the treatment of single-level degenerative cervical spondylosis: a systematic review and meta-analysis
Source: J Orthop Surg Res. 2022 Nov 24;17:506. doi: 10.1186/s13018-022-03387-9 (PMC9694547; doi:10.1186/s13018-022-03387-9)
Supplement: Supplementary file 2 — Additional file 2. Meta-analysis of Zero-p group versus CCP group in preoperative NDI score. [file 13018_2022_3387_MOESM2_ESM.pdf]

	Zero-p			CPC				Mean Difference
Study or Subgroup	Mean	SD	Total	Mean	SD	Total	Weight	IV, Fixed, 95% CI
Avani S. Vaishnav 2018	45.32	19.85	41	37.73	14.39	23	0.3%	7.59 [-0.87, 16.05]
Guangpu Liu 2016	33.35	3.56	31	34.29	3.43	31	7.9%	-0.94 [-2.68, 0.80]
Huaishuan Zhang 2020	35.73	2.81	56	36	3.74	67	17.8%	-0.27 [-1.43, 0.89]
Long Guo 2015	39.5	13.7	49	39.8	13.7	49	0.8%	-0.30 [-5.72, 5.12]
Ning Wang 2016	42.92	1.9	12	42	2.3	16	9.9%	0.92 [-0.64, 2.48]
Ruien Gou 2022	45.1	3.2	16	44.6	3.1	16	5.0%	0.50 [-1.68, 2.68]
Shuangjun He 2021	34.1	9.5	42	33.5	8.9	45	1.6%	0.60 [-3.28, 4.48]
Sung Hyun Noh2021	37.9	1.52	38	38.2	1.75	42	46.5%	-0.30 [-1.02, 0.42]
Yibing Li 2020	19.5	4	24	18.6	4.5	27	4.4%	0.90 [-1.43, 3.23]
Zhidong Wang 2015	24.2	4.1	27	24.9	3.7	30	5.8%	-0.70 [-2.74, 1.34]
**Total (95% CI)**			**336**			**346**	**100.0%**	**-0.11 [-0.60, 0.37]**

Heterogeneity: Chi² = 7.56, df = 9 (P = 0.58); I² = 0%

Test for overall effect: Z = 0.46 (P = 0.65)

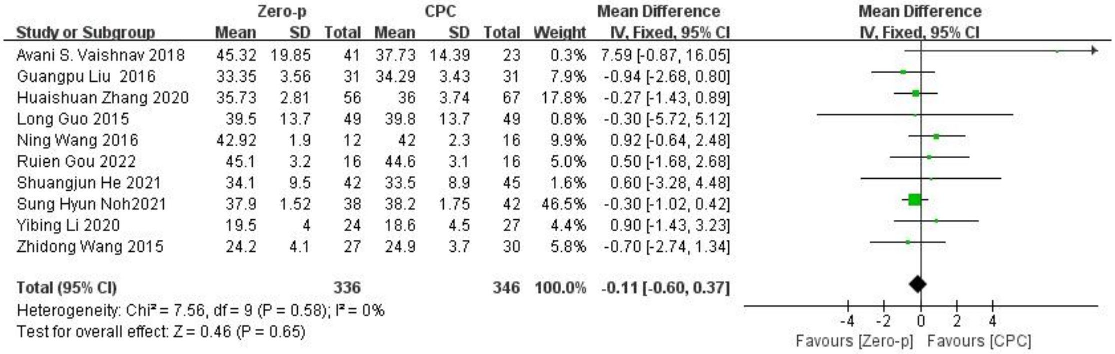

Supplementary material. 2 Meta-analysis of Zero-p group versus CCP group in preoperative NDI score
