# Supplementary material for: Zero-profile implant versus conventional cage–plate construct in anterior cervical discectomy and fusion for the treatment of single-level degenerative cervical spondylosis: a systematic review and meta-analysis
Source: J Orthop Surg Res. 2022 Nov 24;17:506. doi: 10.1186/s13018-022-03387-9 (PMC9694547; doi:10.1186/s13018-022-03387-9)
Supplement: Supplementary file 3 — Additional file 3. Meta-analysis of Zero-p group versus CCP group in preoperative JOA score. [file 13018_2022_3387_MOESM3_ESM.pdf]

Study or Subgroup	Zero-p Mean	Zero-p SD	Zero-p Total	CPC Mean	CPC SD	CPC Total	Weight	Mean Difference IV, Random, 95% CI
Baihan Sun 2017	9.61	0.37	25	10.03	0.24	28	29.8%	-0.42 [-0.59, -0.25]
Buqing Chang 2017	9.33	2.29	21	9.43	2.4	24	1.1%	-0.10 [-1.47, 1.27]
Denglu Yan 2014	8.8	2.6	37	8.4	2.8	35	1.4%	0.40 [-0.85, 1.65]
Guangpu Liu 2016	9.19	2.55	31	9.32	2.62	31	1.3%	-0.13 [-1.42, 1.16]
Haiyu Shao 2016	9.81	2.21	63	9.94	2.35	76	3.6%	-0.13 [-0.89, 0.63]
Huaishuan Zhang 2020	11.43	2.1	56	11.66	1.92	67	4.0%	-0.23 [-0.95, 0.49]
Jun Hu 2017	14.22	0.8	23	14.13	0.67	31	10.7%	0.09 [-0.31, 0.49]
Junpu Luo 2021	9.41	1.31	60	9.37	1.61	60	6.9%	0.04 [-0.49, 0.57]
Jun Zhu 2019	7.95	1.68	19	8.38	1.3	26	2.6%	-0.43 [-1.34, 0.48]
Leixin Wei2022	9.52	0.81	68	9.76	0.76	28	13.7%	-0.24 [-0.58, 0.10]
Liqi Ruan 2018	10	1.8	21	10.9	2.5	18	1.1%	-0.90 [-2.29, 0.49]
Ning Wang 2016	8.42	1.7	12	8.25	1.81	16	1.3%	0.17 [-1.14, 1.48]
Ruien Gou 2022	6.9	1.3	16	7.1	1.2	16	2.8%	-0.20 [-1.07, 0.67]
Shuangjun He 2021	9.9	1.6	42	9.7	1.9	45	3.8%	0.20 [-0.54, 0.94]
Tao lan 2017	9.63	1.48	35	9.21	1.36	33	4.4%	0.42 [-0.26, 1.10]
Weiping Sha 2021	7.93	1.08	30	7.83	1.18	31	6.0%	0.10 [-0.47, 0.67]
Yibing Li 2020	8.1	2.1	24	7.6	3.4	27	0.9%	0.50 [-1.03, 2.03]
Zhidong Wang 2015	8.9	1.1	27	8.7	1.4	30	4.7%	0.20 [-0.45, 0.85]
**Total (95% CI)**			**610**			**622**	**100.0%**	**-0.14 [-0.28, 0.01]**

Heterogeneity: $Tau^2 = 0.01$; $Chi^2 = 19.41$, df = 17 (P = 0.31); $I^2 = 12\%$

Test for overall effect: Z = 1.78 (P = 0.08)

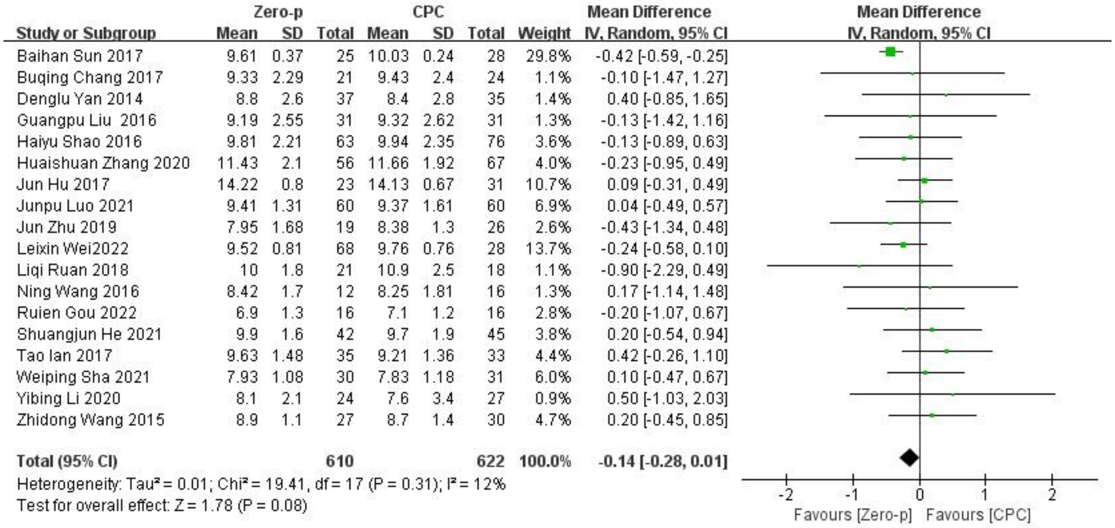

Supplementary material. 3 Meta-analysis of Zero-p group versus CCP group in preoperative JOA score
