# Supplementary material for: Zero-profile implant versus conventional cage–plate construct in anterior cervical discectomy and fusion for the treatment of single-level degenerative cervical spondylosis: a systematic review and meta-analysis
Source: J Orthop Surg Res. 2022 Nov 24;17:506. doi: 10.1186/s13018-022-03387-9 (PMC9694547; doi:10.1186/s13018-022-03387-9)
Supplement: Supplementary file 4 — Additional file 4. Meta-analysis of Zero-p group versus CCP group in preoperative VAS score. [file 13018_2022_3387_MOESM4_ESM.pdf]

	Zero-p			CPC				Mean Difference
Study or Subgroup	Mean	SD	Total	Mean	SD	Total	Weight	IV, Random, 95% CI
Avani S. Vaishnav 2018	6.54	2.24	41	6.19	2.28	23	1.6%	0.35 [-0.81, 1.51]
Baihan Sun 2017	7.21	1.34	25	8.05	2.11	28	2.4%	-0.84 [-1.78, 0.10]
Buqing Chang 2017	6.25	1.49	21	6.21	1.62	24	2.5%	0.04 [-0.87, 0.95]
Denglu Yan 2014	7.67	2.48	37	7.3	2.66	35	1.5%	0.37 [-0.82, 1.56]
Huaishuan Zhang 2020	6.12	2.15	56	5.9	1.92	67	4.0%	0.22 [-0.51, 0.95]
Jun Hu 2017	4.13	0.55	23	4.35	1.23	31	8.8%	-0.22 [-0.71, 0.27]
Junpu Luo 2021	5.21	1.92	60	5.36	1.62	60	5.2%	-0.15 [-0.79, 0.49]
Jun Zhu 2019	6.63	1.12	19	6.34	1.16	26	4.6%	0.29 [-0.38, 0.96]
Leixin Wei2022	4.43	0.88	68	4.16	0.8	28	16.0%	0.27 [-0.09, 0.63]
Osamu Nemoto 2015	4.3	1.4	24	4.5	1.3	22	3.5%	-0.20 [-0.98, 0.58]
Sung Hyun Noh2021	7.9	0.56	38	8.1	0.62	42	31.4%	-0.20 [-0.46, 0.06]
Tao lan 2017	7.23	1	35	7.21	1.14	33	8.1%	0.02 [-0.49, 0.53]
Weiping Sha 2021	4.3	0.95	30	4.13	1.01	31	8.7%	0.17 [-0.32, 0.66]
Yibing Li 2020	7	2.4	24	7.5	1.4	27	1.8%	-0.50 [-1.60, 0.60]
**Total (95% CI)**			**501**			**477**	**100.0%**	**-0.03 [-0.18, 0.11]**

Heterogeneity: Tau² = 0.00; Chi² = 11.61, df = 13 (P = 0.56); I² = 0%

Test for overall effect: Z = 0.43 (P = 0.67)

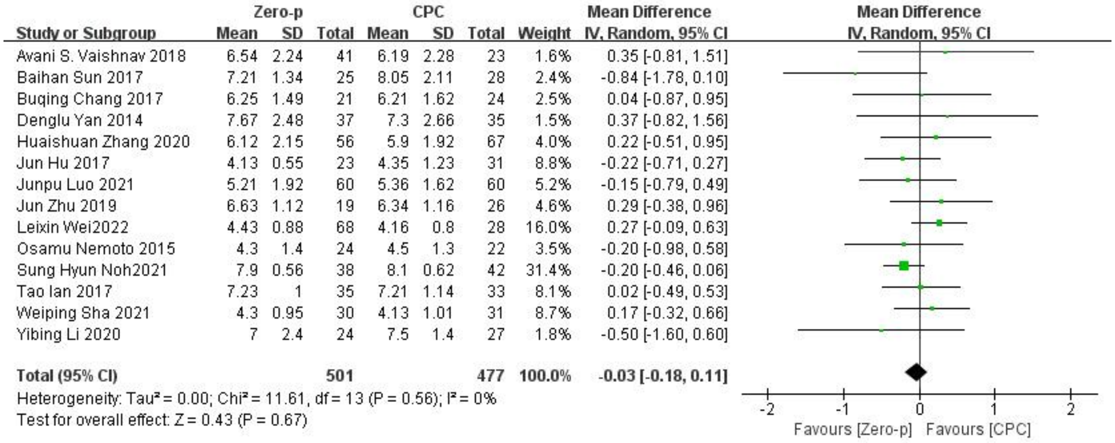

Supplementary material. 4 Meta-analysis of Zero-p group versus CCP group in preoperative VAS score
