# Supplementary figures and images for: Zero-profile implant versus conventional cage–plate construct in anterior cervical discectomy and fusion for the treatment of single-level degenerative cervical spondylosis: a systematic review and meta-analysis
Source: J Orthop Surg Res. 2022 Nov 24;17:506. doi: 10.1186/s13018-022-03387-9 (PMC9694547; doi:10.1186/s13018-022-03387-9)

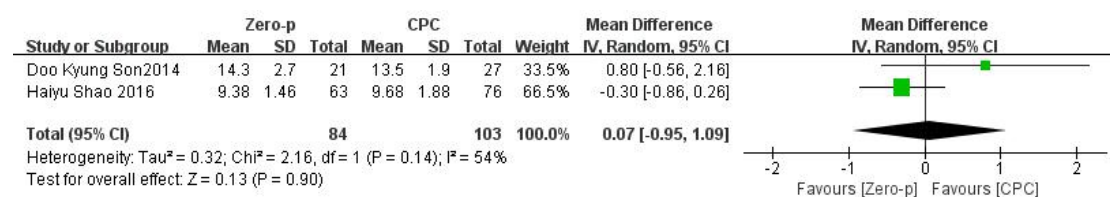

**Supplementary material. 5** Meta-analysis of Zero-p group versus CCP group in preoperative PSTT

Supplement: Supplementary file 5 — Additional file 5. Meta-analysis of Zero-p group versus CCP group in preoperative PSTT. [file 13018_2022_3387_MOESM5_ESM.pdf]
